# G‐Quadruplexes: Structural Diversity and Emerging Roles in Biomolecular Condensation

**DOI:** 10.1002/advs.76663

**Published:** 2026-07-20

**Authors:** Wenmeng Wang, Qingqing Xu, Yuxin Zhang, Fang Liu, Guangchao Sui, Dangdang Li

**Affiliations:** ^1^ College of Life Science Northeast Forestry University Harbin China; ^2^ Department of Medical Oncology Harbin Medical University Cancer Hospital Harbin China

**Keywords:** biomolecular condensates, G‐quadruplexes, multifaceted functions, phase separation

## Abstract

Among non‐classical nucleic acid secondary structures, G‐quadruplexes (G4s) play diverse roles in cellular functions and disease pathogenesis. However, the molecular mechanisms underlying the assembly of endogenous G4s into punctate condensates in cells remain unclear. Biomolecular condensates can arise from weak multivalent intermolecular interactions involving proteins and/or nucleic acids; this phenomenon is frequently linked to liquid–liquid phase separation. Recent research has provided compelling evidence for G4s driving biomolecular condensation. In this review, we first summarize the latest breakthroughs in the structural classification of G4s. In addition to frequently reported intramolecular G4s, intermolecular G4s have also been observed in cellular environments. Next, we discuss the regulatory role of G4s in condensation. Although G4s can independently form condensates, they primarily serve as structural platforms that facilitate condensate formation and regulate their phase transitions. Ultimately, this review reveals the multifaceted physiological and pathological functions of G4‐driven condensates, including chromatin organization, assembly of stress granules and paraspeckles, abnormal transcriptional activation, telomere maintenance, neurodegenerative disease‐associated protein aggregation, and viral inclusion body formation.

## Graphical abstract

G‐quadruplexes (G4s) fold into diverse intra‐ and intermolecular structures, positioning them as emerging regulators of biomolecular condensation. Mechanistically, G4s autonomously form condensates, act as structural platforms to initiate and stimulate condensation, or induce phase transitions. G4‐driven condensates function in chromatin organization, stress granule assembly, and paraspeckle formation. Aberrant G4‐mediated condensation promotes cancer, neurodegeneration, and viral infections.

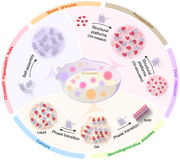

## 1 Introduction

In 1953, Watson and Crick proposed the canonical right‐handed double‐helix structure of DNA, which provides the basis for deciphering the genetic code using information encoded within nucleotide pairing [1]. The composition of each genome sequence determines its specific function, with mutations, deletions, or insertions leading to diseases such as cancer [2, 3, 4]. However, nucleotides may adopt various non‐canonical secondary structural motifs in different cellular environments without changing their sequences. Non‐classical DNA structures identified to date include four‐stranded G‐quadruplexes (G4s), i‐motifs, R‐loops, Z‐DNAs, triplexes, G‐triplexes, G4‐loops, and cruciforms [5, 6, 7, 8, 9, 10, 11]. G4s have attracted considerable attention in chemistry and life sciences because of their distinctive structures and potential roles in diverse biological processes [12].

**FIGURE 1:**
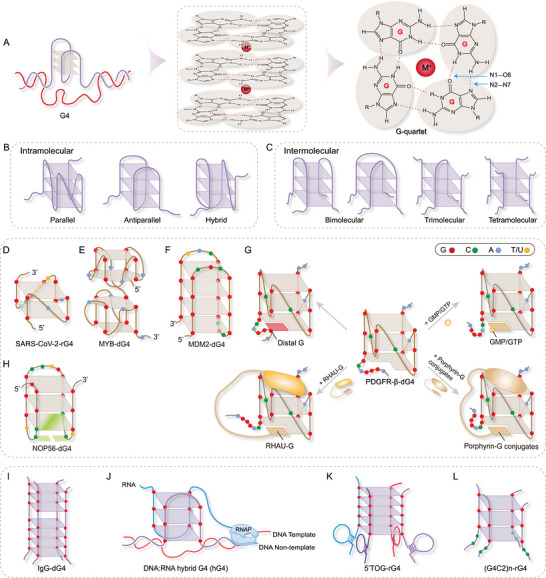
Diagrams of representative G‐quadruplex (G4) structures. (A) Diagrams of a G4 structure and its quartets. A G4 composed of three G‐quartets is presented. Each quartet is formed by four guanines connected through Hoogsteen hydrogen bonds (N1‐O6 and N2‐N7), with a monovalent cation (M^+^, such as K^+^ and Na^+^) located in the center to stabilize the structure. (B) Different topological conformations of intramolecular G4 (intra‐G4) structures. Based on the 5′‐to‐3′ orientation of the four strands in a G4 structure, it can be classified into parallel, antiparallel, or hybrid conformation. (C) Different topological conformations of intermolecular G4 (inter‐G4) structures. Based on the number of DNA molecules contributing to the nucleic acid strands of a G4 structure, it can be classified into unimolecular shown in (B), bimolecular, trimolecular, or tetramolecular conformations. (D) Model of an rG4 formed in the severe acute respiratory syndrome coronavirus 2 (SARS‐CoV‐2) genome. The structure is characterized by the presence of two G‐quartets. Guanines, cytosines, adenines, and thymines/uracils (G, C, A, and T/U) are shown in the figure by red, green, blue, and yellow circles, respectively. The color code is also used in the following diagrams. (E) The G4 in the MYB proto‐oncogene (*MYB*) promoter. The structure contains two G4 motifs, each consisting of two G‐quartets. (F) The mouse double minute 2 homolog (*MDM2*) promoter G4 structure with four G‐quartets. (G) The platelet‐derived growth factor receptor‐β (*PDGFR‐β*) promoter G4. The structure contains three G‐quartets, but a G‐quartet is formed by three guanines. A distal G, along with its derivatives such as GMP and GTP, as well as the RHAU (RNA helicase associated with AU‐rich element) peptide coupled with G (RHAU‐G), and porphyrin‐G conjugates, can fill the plane, leading to the formation of a complete and hybrid G‐quartet. (H) The nucleolar protein 56 (*NOP56*) promoter G4 with two G‐quartets and one C·G·C·G‐quartet. The structure is further capped by a C·C base pair. (I) Model of a tetramolecular dG4 formed by guanine‐rich sequences within the immunoglobulin G (IgG) heavy chain region in vitro. (J) A DNA:RNA hybrid G4 (hG4) formed during transcription by the G‐tracts from both the templated DNA and transcribed RNA. (K) Model of a tetramolecular rG4 with five G‐quartets formed by 5′ terminal oligoguanine (5′TOG) sequences of multiple 5′‐tRNA‐derived small RNA (5′‐tiRNA) fragments. (L) Structure of a tetramolecular rG4 assembled by the (G4C2)_n_ repeat, when n≥8.

G4s are formed in the G‐rich regions of DNA and RNA (dG4s and rG4s, respectively), and typically comprise two or more planar G‐quartet motifs stabilized by π–π stacking. Each G‐quartet is formed by four guanines via Hoogsteen hydrogen bonds (N1‐O6 and N2‐N7). A monovalent cation, typically K^+^, is coordinated at the center of the G‐quartet channel, interacting with eight O6 carbonyl oxygen atoms from two adjacent quartets to mitigate electrostatic repulsion and stabilize the structure (Figure 1A) [12, 13, 14]. Since the discovery of in vitro G4 formation in 1988 [15], genome sequencing analyses and visualization approaches based on antibodies, proteins, and small molecules have convincingly confirmed the presence of G4s in live cells, especially cancer cells [16, 17, 18, 19, 20, 21, 22]. Although G4s exhibit robust functions in various physiological and pathological contexts, the mechanisms regulating these processes remain largely unexplored [12, 23, 24].

Eukaryotic cells contain numerous membrane‐bound organelles–such as the endoplasmic reticulum and mitochondria–and membrane‐less organelles (also called “biomolecular condensates”)–such as the nucleoli and nuclear speckles [25, 26]. The molecular mechanisms underlying the assembly and function of biomolecular condensates are a long‐standing focus of life sciences research. In 2009, Hyman and Brangwynne first observed that P granules, a type of membrane‐less organelle composed of proteins and RNA, exhibited liquid–liquid phase separation (LLPS) characteristics in *Caenorhabditis elegans* germ cells [27]. LLPS has since emerged as a crucial mechanism regulating biomolecular condensate formation in the cellular environment. According to immunofluorescence analyses employing G4‐specific antibodies, endogenous G4 signals exhibit a punctate subcellular distribution in cancer cells [18, 19, 21], which suggests that G4s may form higher‐order assemblies through LLPS and therefore exert regulatory functions in biomolecular condensates.

Recently, several groups have proposed functional roles of G4s (particularly rG4s) in modulating biomolecular condensates, with direct effects on cellular physiology and pathophysiology [28, 29, 30, 31, 32, 33]. Accordingly, clarifying the regulatory relationship between G4s and phase‐separated condensates will provide crucial insights into the key structural roles of G4s in gene regulation and other functions. Here, we review the latest research advances regarding the structural classification of G4s and their regulatory roles in condensate formation and phase transition. Additionally, we highlight the physiological and pathological functions of condensates regulated by G4s.

## 2 DNA or RNA Sequences Fold Into Various G4 Conformations

Unlike double‐helical DNA structures, G4 nucleic acid sequences have various base compositions and lengths, resulting in diverse topologies. G4s can fold into parallel, antiparallel, and hybrid conformations depending on the relative orientations of the folding nucleic acid strands (Figure 1B) [14, 34]. rG4s assemble more readily into a parallel conformation than dG4s owing to the presence of 2′‐hydroxyl groups in RNA, which form additional hydrogen bonds that ensure a rigid and uniform backbone geometry and favor a parallel topology [35, 36]. Without these groups, DNA exhibits greater backbone flexibility and the ability to adopt multiple topologies [14, 37, 38]. Thus, rG4s exhibit higher thermodynamic stability (typical melting temperature 5°C–15°C higher than their DNA counterparts under the same ionic conditions), lower hydration, and greater compactness than dG4s [37, 38, 39, 40].

Additionally, according to the number of nucleic acid molecules involved in structure formation, G4s may have intramolecular (intra‐G4s) or intermolecular (inter‐G4s) conformations (Figure 1B,C) [14, 34], which exhibit distinct chemical and biophysical properties. Intra‐G4s form from a single strand with capping loops that shield the terminal G‐quartets, whereas inter‐G4s assemble from multiple strands without such loops, leaving their ends exposed and creating multivalent binding interfaces [12, 41]. Intra‐G4s fold rapidly in the presence of both K^+^ and Na^+^ and exhibit variable thermal stability (typical melting temperatures 40°C–70°C under physiological K^+^ concentrations), with K^+^ promoting more stable structures [42, 43]. Inter‐G4s, particularly tetramolecular species, fold more slowly and display higher thermal stability (melting temperature > 80 °C) owing to uninterrupted G‐quartet stacks, requiring K^+^ rather than Na^+^ for optimal formation [42, 43]. Thus, intra‐G4s appear better suited for dynamic regulatory roles, whereas inter‐G4s may function primarily in stable structural contexts. In this section, we review recent research on the structural features of G4s, particularly inter‐G4s.

### 2.1 Intramolecular G4 Structures (Intra‐G4s)

Intra‐G4s represent the dominant structural type in living cells [34]. The conserved motif is commonly described as G_≥2_N_1‐7_G_≥2_N_1‐7_G_≥2_N_1‐7_G_≥2_, where N represents A, G, C, and T or U [34, 44]. Loop lengths greater than seven nucleotides (>7 nt) can reduce stability without entirely preventing folding; however, exceptionally long loops (typically >13 nt) may inhibit G4 formation in specific sequence contexts. Most reported intra‐G4s comprise three G‐quartets, including dG4s formed in MYC proto‐oncogene (*MYC*) or vascular endothelial growth factor promoter regions or in telomeres, and rG4s formed in B‐cell lymphoma 2 (*BCL2*) mRNA [45, 46, 47, 48]. rG4s are more prone to assume conformations composed of two G‐quartets. For instance, the genome of the 2019 novel coronavirus can form rG4s consisting of two G‐quartets (Figure 1D) [49]. Importantly, these rG4s exhibit increased stability [50]. Similarly, Matsugami et al. first reported a novel dG4 structure formed by multiples GGA repeats in the MYB proto‐oncogene (*MYB*) promoter, which exhibits a tetrad:heptad:heptad:tetrad (T:H:H:T) arrangement by stacking two T:H G4s; within this dG4 structure, two dG4s create a dimer stabilized by stacking interactions between their heptads (Figure 1E) [51]. In addition, Lago et al. reported dG4s comprising four G‐quartets in the mouse double minute 2 homolog promoter (Figure 1F) [52]. As a greater number of G‐quartets can enhance G4 stability and structural specificity [53], the potential for intra‐G4s comprising five or more G‐quartets in the cellular environment should also be explored.

Interestingly, the platelet‐derived growth factor receptor‐β gene promoter forms a G‐vacancy G4 structure, which is primarily characterized by one plane comprising three guanines [54, 55, 56]. The gap in this plane can be filled by the distal G to form a complete G‐quartet [55, 56], or by derivatives of G, including GMP and GTP, the RHAU (RNA helicase associated with AU‐rich element) peptide coupled with G (RHAU‐G, which can bind G4s), or porphyrin‐G conjugates (Figure 1G) [54, 57, 58]. Importantly, G4s with G‐vacancies are prevalent in promoter regions [57, 58, 59]. In addition to G‐vacant G4s, Yan et al. recently discovered a 21‐nucleotide sequence from intron 1 of the nucleolar protein 56 gene that can fold into an antiparallel chair‐type dG4 structure [60]. This structure contains two G‐quartets and one C·G·C·G‐quartet, which is additionally capped by a C·C base pair (Figure 1H) [60]. Thus, nucleic acid sequences that deviate from the conventional sequence formula for intra‐G4 structures can form distinct types of G4s, which pose challenges for many G4 prediction algorithms. Therefore, G4 prediction software should be integrated with additional techniques, such as chromatin immunoprecipitation sequencing assays using the BG4 antibody, which specifically recognizes G4s [17].

### 2.2 Intermolecular G4 Structures (Inter‐G4s)

Inter‐G4s are formed by two to four nucleic acid molecules, primarily in bimolecular, trimolecular, and tetramolecular conformations (Figure 1C) [14, 34]. Current research on the biological roles of G4s generally focuses on intra‐G4s owing to the low probability of inter‐G4 formations occurring in genomic DNA under cellular conditions. Many previous studies have indicated that inter‐G4s only form in vitro, such as the first discovery of G4s formed by G‐rich sequences within the immunoglobulin G (IgG) heavy chain region (Figure 1I) [15]. However, other researchers have demonstrated the existence of inter‐G4s *in cellulo*.

First, Wu et al. reported the formation of a specific G4 type called DNA:RNA hybrid G4s (hG4s), located within transcribed plasmids in bacteria [61]. This unique conformation forms through the concurrent gathering of G‐tracts from a non‐template DNA strand and RNA transcripts (Figure 1J) [61]. More recently, hG4s were observed in the genomes of living eukaryotic cells [62]. hG4s benefit from easy assembly and often take precedence at G4 sites, showing the potential to create intra‐dG4s independently. In contrast to dG4s, hG4s exhibit enhanced structural diversity and stability, and their formation is closely associated with transcription [62]. In summary, dominant and genome‐wide hG4 formation may be common in both prokaryotic and eukaryotic cells.

Second, tRNA‐derived stress‐induced RNAs (tiRNAs) are primarily produced through cleavage of the anticodon loops of mature tRNAs by the ribonuclease angiogenin under various stressors, such as oxidative stress and ultraviolet irradiation [63, 64]. tiRNAs include 5′‐ and 3′‐tiRNAs, with 5′‐tiRNAs capable of inhibiting translation initiation and triggering the assembly of stress granules (SGs); this process is dependent on a 5′ terminal oligoguanine (5′TOG) [63, 64]. In 2017, Lyons et al. reported that the 5′TOG sequences of multiple 5′‐tiRNA fragments can assemble into unique and stable tetramolecular rG4s containing five G‐quartets (Figure 1K) [63]. Notably, inter‐rG4 disruption eliminates the capacity of 5′‐tiRNAs to trigger SG formation [63], underscoring the functional significance of inter‐rG4s.

Third, the primary genetic factor underlying amyotrophic lateral sclerosis (ALS) and frontotemporal dementia (FTD) is an expansion of the hexanucleotide repeat sequence (G4C2)_n_ located within the intron of the chromosome 9 open reading frame 72 (*C9orf72*) gene [65, 66]. Recently, Raguseo et al. employed various biophysical techniques to demonstrate that the RNA (G4C2)_n_ repeat can readily assemble into tetramolecular inter‐rG4s under physiological conditions when n ≥ 8 (Figure 1L) [67]. Compared with healthy controls, these rG4 signals were significantly increased in *C9orf72* mutant motor neurons, as evidenced by the application of the G4‐selective fluorescent probe N‐methyl mesoporphyrin IX (NMM) [67]. Thus, inter‐rG4 structures also form in ALS/FTD‐affected cells.

In addition, the Antonio group reported the first example of an endogenous protein, Cockayne Syndrome B (CSB), which exhibits high affinity and specificity for inter‐G4s formed within ribosomal DNA but negligible binding to intra‐G4s [68]. Pronounced BG4 immunostaining in the nucleoli of CSB‐deficient cells was reduced by CSB re‐expression [68]. As dense regions of the nucleus containing highly G‐rich ribosomal DNA [69, 70], nucleoli represent an optimal environment for inter‐G4 formation. Thus, these observations imply that inter‐G4s assemble within ribosomal DNA and provide functional binding sites for CSB.

The identification of CSB as an inter‐G4‐binding protein implies that other proteins may also recognize such structures. Various chromatin components within the nucleus interact with chromatin structural proteins to facilitate close connections, inducing or repressing gene expression [71, 72]. Therefore, distinct chromatin molecules may also form inter‐G4s; the interactions among different chromatin molecules mediated by this structure may represent a significant mechanism for regulating transcription. Furthermore, specific chromatin structural proteins within the nucleus may possess the ability to bind inter‐G4s. Future studies should develop tools–such as peptides derived from inter‐G4‐binding domains–to detect these structures within the cellular environment.

## 3 G4s as Emerging Regulators of Condensate Formation and Phase Transitions

**FIGURE 2:**
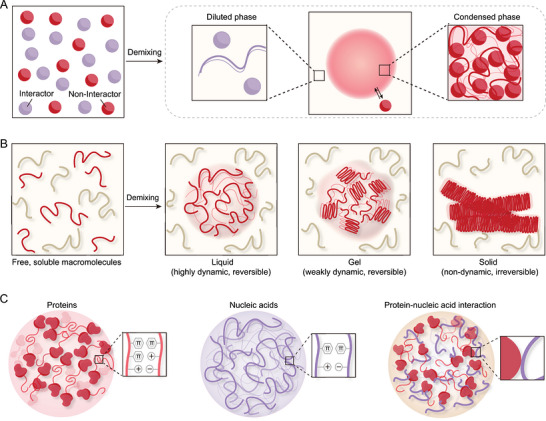
General principles of phase‐separated condensate formation. (A) Diagram of basic phase‐separated condensation. In a solution containing different biomolecules, condensates are formed through molecule–molecule interactions and repulsions, leading to the demixing of two distinct phases: condensed and diluted. In the condensed phase, proteins with suitable or compatible properties are enriched, while other molecules are effectively excluded. (B) Different states of phase separation. Condensates primarily encompass three physical states: liquid, gel, and solid. Among them, liquid condensates exhibit the highest molecular dynamics, while gel condensates exhibit relatively lower molecular dynamics. However, solid condensates completely lack molecular motility. (C) Different interactions among molecules in condensates. The weak interactions among intrinsically disordered regions (IDRs) (left), the electrostatic interactions between nucleic acids (middle), and the direct binding of proteins to nucleic acids (right) facilitate the formation of condensates via liquid–liquid phase separation (LLPS).

Phase separation is conventionally defined as the spontaneous division of a homogeneous solution into two distinct phases: condensed and diluted (Figure 2A). The condensed phase is often referred to as “condensates” (termed “biomolecular condensates” in the Introduction) [26, 73]. In this review, we use the term “condensation” to describe the condensate formation process. Condensates exist in three common material states: liquid, gel, and solid (Figure 2B) [25, 74]. Liquid condensates are highly dynamic assemblies formed via LLPS, with components in the droplets capable of exchanging with the surrounding environment. Gel condensates exhibited reduced dynamics, allowing only limited exchange of components. Solid condensates lose their dynamic nature, rendering their internal components immobile (Figure 2B) [25, 74]. As well as molecular mobility differences, these states exhibit significant variations in their physical properties, such as viscosity, elasticity, and surface tension [26, 75]. Phase transition refers to the transformation between distinct material states, such as the water‐to‐ice transition [25, 74]. In this review, when referring to specific examples, we describe these states as “liquid‐like,” “gel‐like,” or “solid‐like” (see Note S1 for experimental guidelines) to reflect the fact that experimental observations often approximate, but may not perfectly match, ideal material states.

Biomolecular condensates primarily form through the phase separation of intrinsically disordered region (IDR)‐containing proteins and/or nucleic acids (Figure 2C) [26, 76]. The protein component is generally considered the primary trigger for phase separation, with less focus on the key contribution of nucleic acids to this process. However, ample evidence has confirmed that RNAs, such as long non‐coding and circular RNAs, double‐stranded DNAs (dsDNAs), and various non‐classical secondary structures of nucleic acids, including G4s, R‐loops, and i‐motifs, serve as important regulators of biomolecular condensation [77, 78, 79, 80, 81, 82].

**FIGURE 3:**
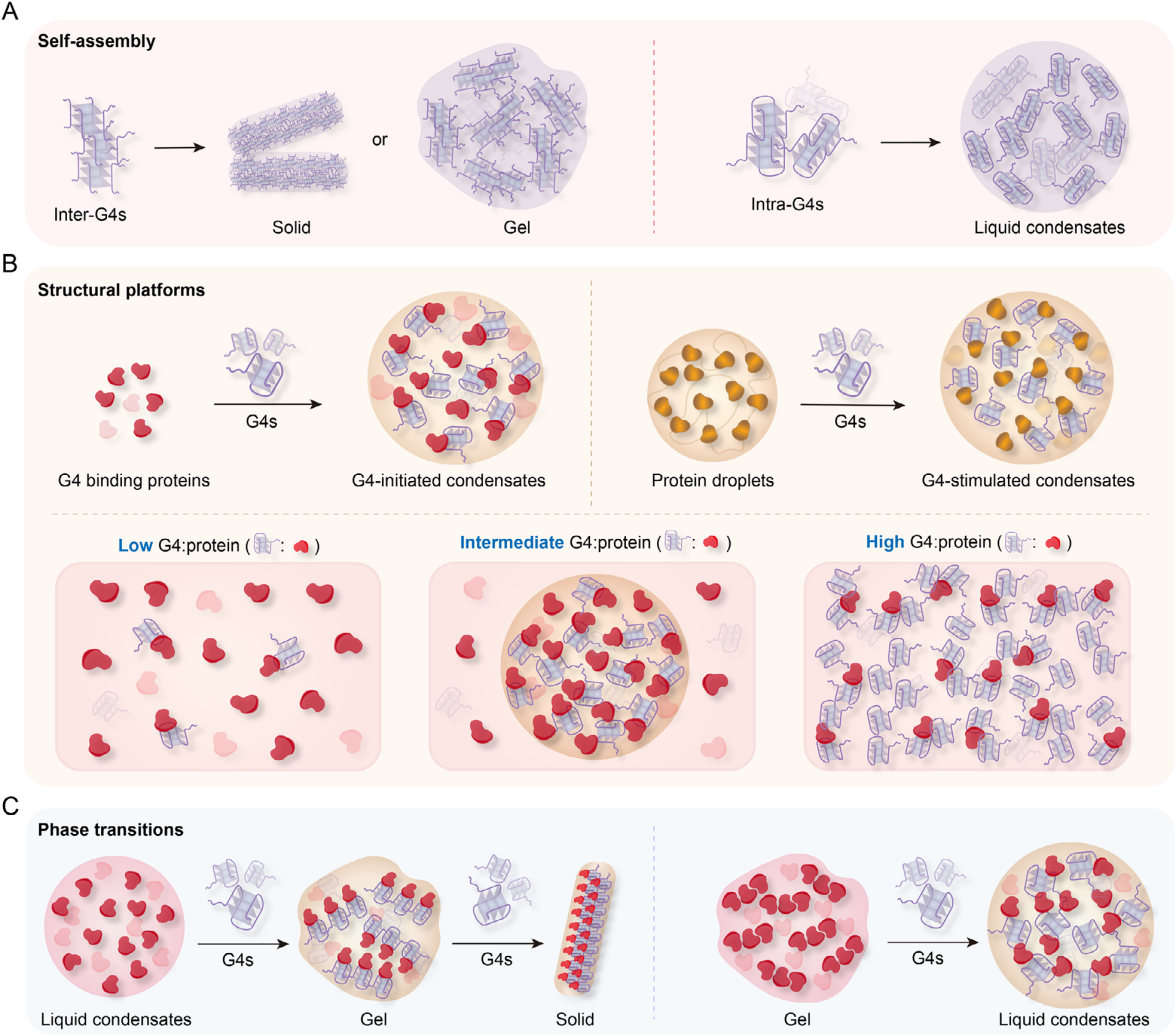
Regulatory mechanisms of G‐quadruplexes (G4s) in condensation. (A) Condensates formed by self‐assembly of G4 molecules. A large amount of intermolecular G4s (inter‐G4s) facilitates the formation of gel or solid condensates by themselves (left), whereas intramolecular G4s (intra‐G4s) generally generate liquid condensates (right). (B) G4s serve as structural platforms that initiate and stimulate condensation. In G4‐initiated condensates, proteins alone are unable to start the condensation process; however, the addition of G4s enables proteins to form condensates (top left). Conversely, in G4‐stimulated condensation, proteins are capable of undergoing liquid–liquid phase separation (LLPS) independently; the addition of G4s enhances the condensation to form larger condensates (top right). Importantly, an appropriate concentration of G4s can drive protein LLPS, while lower G4 concentrations fail to drive protein condensation, and higher concentrations disrupt the condensates (bottom). (C) Diagrams of G4‐regulated phase transition. G4s can promote the transition of condensates from a liquid state to a gel or solid state (left). Additionally, G4s are also capable of facilitating the gel‐to‐liquid transition of condensates (right).

In this section, we discuss the emerging roles of G4s in the formation and phase transition of condensates within a clear conceptual framework (Figure 3A–C). Specifically, we organize the literature on G4‐modulated condensation into three mechanistic dimensions to elucidate the distinct mechanisms by which G4s regulate condensation. Section 3.1 focuses on G4‐driven condensation without protein involvement, particularly intra‐G4 (liquid‐like) versus inter‐G4 (gel/solid‐like). Section 3.2 addresses G4s as structural platforms, specifically G4s that provide multivalent binding surfaces for organizing protein condensation. This section further differentiates between G4‐initiated mechanisms (where G4s are indispensable for proteins to undergo LLPS) and G4‐stimulated mechanisms (where protein‐autonomous LLPS is enhanced by G4s). Section 3.3 discusses the G4‐regulated phase transitions between liquid‐, gel‐, and solid‐like states. These three categories are not mutually exclusive but rather represent a mechanistic framework for understanding G4‐dependent condensation from different perspectives. The findings presented in this review are based on a growing yet limited body of evidence; therefore, a critical discussion of the limitations and remaining questions in the field is provided in Section 5. Table 1 provides a comprehensive overview of the G4‐driven condensation examples discussed in this review, including their mechanistic categories, G4 types, G4 sources, protein partners, location (cellular compartments), evidence types, material states (experimental verification), and functional outcomes.

**TABLE 1:** A structured atlas of G4‐driven condensation: Classification from mechanistic categories to functional outcomes.

Mechanistic categories	G4 types	G4 sources	Protein partners	Location (cellular compartments)	Evidence types	Material states (experimental verification)	Functional outcomes	Ref.
**G4 self‐assembly**	Inter‐rG4	*C9orf72* RNA (G4C2)n repeats	None	Nucleus (Nuclear speckles)	In vitro/*in cellulo*	Gel‐like (Morphological observation, FRAP)	ALS/FTD	[87]
				Nucleus	In vitro/*in cellulo*	Gel‐like (Morphological observation, TEM)	Drives TDP‐43 aggregation, putative role in ALS/FTD	[67]
	Inter‐rG4	Small RNAs (e.g., GCGGCGGC, CCGGGGCC)	None	Nucleus	In vitro/*in cellulo/*in vivo	Solid‐like (FRAP, NMR, Fluorescence imaging)	Neurological diseases	[86]
	Inter‐rG4	Poly(UG) repeats	None	In vitro only	In vitro	Gel‐like (FRAP)	Putative role in gene silencing	[84]
	Intra‐rG4	*SHR* mRNA G4	None	Cytoplasm (Mainly)	In vitro/*in cellulo*	Liquid‐like (Fusion)	Putative role in plant root development	[90]
	Intra‐rG4	*Mark2* mRNA G4	None	In vitro only	In vitro	Liquid‐like (FRAP)	Promotes DNAPTP6 LLPS, contributes to stress granule assembly	[29]
	Intra‐rG4	TERRA‐rG4	None	In vitro only	In vitro	Liquid‐like (Fusion, FRAP)	Promotes α‐syn aggregation, contributes to PD	[31]
**Structural platform (G4‐initiated)**	Intra‐dG4	G4‐forming DNAs (e.g., *MYC*, telomeric dG4)	H1	Nucleus (Heterochromatin)	In vitro	Liquid‐like (Fusion, FRAP)	Putative role in heterochromatin formation and gene silencing	[82]
	Intra‐dG4	*KRAS* promoter G4	HMGB1	In vitro only	In vitro	Liquid‐like (Fusion, FRAP)	Putative role in transcriptional regulation	[104]
	Intra‐dG4	G4‐forming DNAs (e.g., T95‐2T, CEB1, HT G4)	RECQ4	In vitro only	In vitro	Liquid‐like (NMR, Stopped‐flow assay)	Not determined	[105]
	Intra‐dG4	*MYC*, *BCL2* promoter G4	ZNF706	Nucleus (Transcriptional centers)	In vitro/*in cellulo*	Liquid‐like (Fusion, FRAP)	Putative role in transcriptional regulation	[106]
	Intra‐dG4	Super‐enhancer G4	BRD4	Nucleus (Transcriptional centers)	In vitro	Liquid‐like (Fusion, FRAP, AFM)	Transcriptional activation (indirect evidence)	[107]
**Structural platform** **(G4‐stimulated)**	Intra‐dG4	d(G3A)_4_	SERBP1	In giant membrane vesicles only	In giant membrane vesicles	Liquid‐like (Reversible assay)	Not determined	[109]
	Intra‐dG4	dG4 in cccDNA of HBV	FUS	Nucleus (Transcriptional centers)	*In cellulo*	Liquid‐like (FAPS)	Promotes cccDNA transcription and HBV replication	[110]
	Intra‐dG4	*BCL3* promoter G4	SP1	Nucleus (Transcriptional centers)	In vitro/*in cellulo*	Liquid‐like (Fusion, FRAP)	Transcriptional activation	[111]
	Intra‐rG4	G4‐forming RNAs (e.g., TERRA‐rG4)	SERF2	Cytoplasm (Stress granules)	In vitro/*in cellulo*	Liquid‐like (FRAP, Single‐molecule tracking, Optical tweezers)	Putative role in stress granule assembly	[108]
	Intra‐rG4	Host (MRPS34 mRNA) rG4	Phosphoprotein (P)	Cytoplasm (Inclusion bodies)	In vitro	Liquid‐like (Fusion, FRAP)	Genome replication and MuV infection	[113]
	Intra‐rG4	ribosomal RNA G4	APE1	Nucleolus	In vitro	Liquid‐like (Morphological observation)	Putative role in ribosomal RNA transcription	[114]
	Intra‐rG4	Mark2 mRNA G4	DNAPTP6	Cytoplasm (Stress granules)	In vitro/*in cellulo*	Liquid‐like (FRAP)	Stress granule assembly	[29]
	Intra‐rG4	TERRA‐rG4	LSD1	Nucleus (Telomeres)	In vitro/*in cellulo*	Liquid‐like (Fusion, FRAP)	Telomere maintenance	[116]
**G4‐regulated phase transition**	Intra‐dG4	*CCND1* promoter G4	MAZ	Nucleus (Transcriptional centers)	In vitro/*in cellulo*	Gel‐to‐liquid transition (Fusion, FRAP)	Transcriptional activation and liver cancer development	[28]
	Inter‐rG4	r(G4C2) repeats	TDP‐43	In vitro only	In vitro	Liquid‐to‐solid transition (Morphological observation)	Putative role in ALS/FTD	[67]
	Intra‐rG4	r(G4C2)_4_	FUS	In vitro only	In vitro	Liquid‐to‐solid transition (Fusion, TEM)	Putative role in ALS/FTD	[112]
	Intra‐rG4	CGG repeat‐derived rG4	FMRpolyG	Cytoplasm and nuclear granules	In vitro/in vivo	Liquid‐to‐solid transition (FRAP, TEM, Fluorescence imaging)	FXTAS	[30]
	Intra‐rG4	UGA(GGU)_2_A(GGU)_2_AA	poly‐L‐lysine	In vitro only	In vitro	Liquid‐to‐solid transition (FRAP, CP‐AFM)	Not determined	[119]
	Intra‐rG4	TERRA‐rG4	α‐syn	Cytoplasm	In vitro/*in celllulo/*in vivo	Liquid‐to‐solid transition (FRAP)	PD	[31]
	Intra‐rG4	r(UUAGGG)_4_	Tau	Cytoplasm	In vitro/*in celllulo*	Liquid‐to‐solid transition (FRAP, TEM)	Putative role in AD	[120]

Examples in this table are organized primarily by mechanistic category and secondarily by order of appearance in the main text. For the CCND1‐dG4/MAZ and TERRA‐rG4/α‐syn condensates, G4 both promotes phase transition (shown in the table) and stimulates condensate formation (not shown). Abbreviations used in this table that are not defined in the main text: MRPS34, mitochondrial ribosomal protein S34; TEM, transmission electron microscopy; AFM, atomic force microscopy; FAPS, fluorescence‐activated particle sorting; CP‐AFM, colloidal probe atomic force microscopy. For all other abbreviations, please refer to the main text.

### 3.1 G4 Molecules Alone Drive Condensate Formation

In contrast to DNA, RNA molecules possess the intrinsic ability to form base pairs and create complex secondary and tertiary structures [83]. However, G4 structures are more three‐dimensional and stable than RNAs and display extensive base stacking and hydrogen bonding [84]. Moreover, G‐quartets at the ends of different G4s are more likely to self‐organize into higher‐order structures through strong end‐to‐end stacking interactions facilitated by their wider surface areas (Figure 3A) [85, 86]. These characteristics increase the likelihood of a crowded molecular environment that potentiates the self‐assembly of rG4s into condensates (Figure 3A). For example, ALS/FTD‐associated (G4C2)_n_ repeats can assemble rG4s in *C9orf72* to form gel‐like condensates both in vitro and *in cellulo* [87]. Similarly, (G4C2)_n_ repeats can generate protein‐free gel‐like condensates sustained by their capacity to form tetramolecular inter‐rG4s [67].

As well as G4s formed by long RNA sequences, those formed by short RNAs can also trigger phase separation. Small RNAs such as GCGGCGGC and CCGGGGCC can self‐aggregate into solid‐like condensates via inter‐rG4 structures; distinct condensates formed by these short RNAs have been observed both *in cellulo* and in vivo [86]. Poly(UG) dinucleotide repeats (pUG) are highly abundant in eukaryotic RNAs and have been implicated in gene silencing [88, 89]. Recently, circular dichroism spectroscopy revealed that short pUGs with 3–5 repeats formed inter‐rG4s [84]. These pUGs efficiently self‐assemble into gel‐like condensates via inter‐rG4 interactions, and their ability to form condensates depends on an increase in K^+^ concentration [84].

In conclusion, the condensates formed by inter‐rG4s exhibit poor molecular dynamics and gel‐like behavior, as observed for both *c9orf72* (G4C2)n repeats and pUG‐repeat RNAs. Mechanistically, both repetitive sequences promoted inter‐rG4 formation, which may anchor multiple nucleic acid strands and restrict their mobility within condensates. This restriction reduces internal dynamics, favoring a gel‐like phase over a liquid state. Biologically, the significance of gel‐like RNA condensates appears to be context‐dependent. In the context of *c9orf72* ALS/FTD, persistent gel‐like assemblies of (G4C2)n repeats may contribute to pathological RNA aggregation, impaired nucleocytoplasmic transport, and cellular toxicity [67]. Conversely, pUG repeat condensates in eukaryotes have been implicated in gene silencing [84, 89], suggesting that gel‐like RNA assemblies have physiological functions. Therefore, the gel‐like state represents a common biophysical endpoint with divergent functional outcomes according to the cellular context.

Given that inter‐rG4 formation leads to gel‐ or solid‐like aggregates with restricted molecular dynamics, intra‐rG4‐driven condensates may exhibit superior molecular dynamics to their inter‐rG4 counterparts. This hypothesis is supported by several published studies. First, Zhang et al. demonstrated that *SHORT ROOT* (*SHR*) mRNA formed intra‐rG4s comprising two G‐quartets, which subsequently induced RNA‐driven condensation under physiological conditions [90]. SHR‐rG4‐triggered condensates underwent rapid rearrangement and fusion events, indicating liquid‐like properties. Additionally, the extent of rG4‐triggered LLPS increases with the number of G‐quartets and rG4 loop length [90], confirming the potential stimulatory role of rG4s in the condensation of *SHR* mRNA. Second, Asamitsu et al. discovered that a G‐rich sequence located in the 3′‐untranslated region of microtubule affinity‐regulating kinase 2 (*Mark2*) mRNA can adopt intra‐rG4s containing three G‐quartets [29]. Furthermore, the Mark2‐intra‐rG4s alone could form large spherical droplets with strong dynamic properties, with droplet formation dependent on RNA concentration [29], indicating that intermolecular interactions play a crucial role in facilitating effective LLPS of this RNA.

More recently, Matsuo et al. confirmed that intra‐rG4s formed by telomeric repeat‐containing RNA (TERRA) (UUAGGG)_4_ repeats can undergo LLPS in vitro, even in the absence of polyethylene glycol (PEG) and proteins. Although this process is relatively inefficient, it can be enhanced electrostatically by the presence of Ca^2+^, a divalent cation that accumulates intracellularly during neurodegeneration [31]. However, TERRA condensation is highly context‐dependent, influenced by factors such as RNA concentration and buffer composition. Furthermore, stable G4 formation does not necessarily confer phase‐separation capability; G4s may promote condensation only under specific favorable conditions. Indeed, biophysical studies using unlabeled TERRA have reported non‐phase‐separated states even at concentrations reaching tens of micromolar [91, 92, 93], indicating that TERRA does not always undergo autonomous LLPS under all conditions. Nevertheless, we acknowledge that while TERRA rG4s can undergo LLPS in vitro, TERRA RNA is generally highly soluble under physiological conditions [91, 94], and autonomous LLPS has not yet been demonstrated at endogenous TERRA levels *in cellulo*. Therefore, the condensation of TERRA rG4s is a conditionally regulated process, rather than an intrinsic property.

In summary, condensates formed by intra‐rG4s exhibit notably more robust molecular dynamics than those formed by inter‐rG4s. However, the molecular mechanisms underlying intra‐rG4 and inter‐rG4 self‐assembly into condensates require further investigation. We speculate that these differential dynamics arise from differences in the valence topology and network architecture. Inter‐rG4s function as multivalent cross‐linkers by tethering distinct strands to a spatially constrained network; this configuration restricts molecular diffusion and drives the system toward gelation or solidification. Conversely, intra‐rG4s maintain flexible single‐stranded linkers between folded G4 units; this enables them to act as molecular hinges to facilitate internal rearrangement, droplet fusion, and rapid component exchange, collectively promoting dynamic liquid‐like condensation.

Furthermore, rG4s can spontaneously undergo phase separation, forming condensates at relatively low concentrations, whereas dG4s require higher concentrations or the presence of additional factors–such as proteins or multiple cationic amino acids–to mitigate electrostatic repulsion and promote condensation [95, 96]. This distinction may stem from the unique properties inherent to rG4s. First, rG4s adopt a uniform parallel topology characterized by consistent G‐quartet stacking, whereas dG4s exhibit topological polymorphism. The structural homogeneity of rG4s facilitates more regular intermolecular stacking, reducing entropic penalties. Second, the 2'‐hydroxyl group of ribose–lacking in dG4s–enhances hydrogen‐bonding capacity, functioning as an intrinsic adhesive that promotes interactions among individual rG4s [97].

### 3.2 G4s Act as Structural Platforms to Initiate and Stimulate Condensate Formation

Although G4s can undergo condensation, many biological condensates arise from interactions between G4s and proteins *in cellulo*. Owing to their effective binding affinity to various proteins, including transcription factors, RNA‐binding proteins, and helicases [24, 98, 99, 100, 101, 102, 103], G4s may represent robust structural platforms for condensate assembly (Figure 3B). Mechanistically, G4s provide a multivalent binding surface capable of simultaneously engaging multiple protein molecules, particularly those containing arginine‐rich motifs (RGG/RG repeats) that form cation‐π interactions with G‐quartets. Through this mechanism, G4s attract various types and numbers of proteins, enhance their local enrichment, and lower the effective concentration required for phase separation, thereby initiating and stimulating condensation (Figure 3B, top).

G4s have a known role as structural platforms in condensate formation. First, G4s can directly initiate condensate formation. In G4‐initiated condensates, the proteins alone cannot undergo LLPS; however, the addition of G4s can trigger *de novo* LLPS (Figure 3B, top left). In 2021, Mimura et al. provided the first evidence that both parallel *MYC* promoter dG4s and antiparallel telomeric dG4s serve as structural components that recruit histone H1, initiating G4/H1 condensate formation in vitro [82]. These condensates are likely formed not only through electrostatic interactions between anionic ssDNA and the cationic IDR of H1, but also through π–π stacking interactions among G4s [82]. Subsequently, Wang et al. demonstrated that the parallel dG4 structure in the Kirsten rat sarcoma viral oncogene homolog (*KRAS*) promoter (KRAS‐dG4) and the high‐mobility group protein 1 (HMGB1) assemble into condensates through a mutually reinforcing mechanism, yet are incapable of LLPS independently [104]. Mechanistically, KRAS‐dG4 interacts with HMGB1 to form a stable KRAS‐dG4/HMGB1 complex, which also stabilizes dG4s. The nonspecific interaction between the KRAS‐dG4/HMGB1 complex and additional HMGB1 enhances LLPS. However, the introduction of KRAS‐dG4 or excess K^+^ can inhibit condensate formation, indicating that a balance between HMGB1 and KRAS‐dG4 is essential for co‐condensation [104]. Recent evidence increasingly shows that various types of dG4s drive the condensate formation of proteins such as RecQ protein‐like 4 (RECQ4), zinc factor 706 (ZNF706), small EDRK‐rich factor 2 (SERF2), and low concentrations of bromodomain‐containing protein 4 (BRD4), which cannot undergo LLPS alone [105, 106, 107, 108].

Unlike in G4‐initiated condensates, in G4‐stimulated condensates, proteins can autonomously undergo LLPS, yet the introduction of G4s enhances the LLPS process (Figure 3B, top right). Liu et al. first used giant membrane vesicles as a protocell model, demonstrating that parallel dG4 structures and the RNA‐binding protein SERBP1 (SERPINE1 mRNA binding protein 1) functionally cooperate to form condensates [109]. Moreover, parallel dG4s, rather than other dG4 types, facilitate SERBP1 LLPS in vitro, which can be attributed to their specific binding to SERBP1 [109]. dG4s also played an enhanced role in LLPS of the fused in sarcoma (FUS) protein, and multiple dG4s developed within the covalently closed circular DNA (cccDNA) of hepatitis B virus (HBV) [110]. Notably, deletion of the G4‐binding domain of RGG from FUS led to notably decreased condensation in HBV‐infected HepG2 cells [110]. Similarly, our group provided convincing evidence that various dG4s play a crucial role in augmenting the LLPS ability of the transcription factor MYC‐associated zinc finger (MAZ) in vitro [28]. MAZ can bind to various dG4s, and dG4s containing a higher number of G‐quartets and longer loops exhibit a stronger capacity to stimulate MAZ LLPS in vitro [28]. A more recent study reported that dG4s enhance LLPS for another G4‐binding transcription factor, specificity protein 1 (SP1) [111]. Thus, dG4s are important structural components that promote the condensation of G4‐binding proteins.

Since Ishiguro et al. reported that rG4s can bind FUS and promote LLPS [112], many studies have emphasized the specific structural role of rG4s in aiding condensate formation. For example, rG4s serve as structural platforms that enhance the condensation of P proteins, which are central to the assembly of inclusion bodies for the mumps virus [113]. rG4s are important structural elements that promote the formation of apurinic/apyrimidinic endodeoxyribonuclease 1 (APE1) condensates in the nucleolus [114]. Additionally, two studies have shown that rG4s promote SG assembly by interacting with the SG marker protein Ras GTPase‐activating protein‐binding protein 1 (G3BP1) or the rG4‐binding protein DNA polymerase‐transactivated protein 6 (DNAPTP6) [29, 115]. In Mark2‐rG4‐promoted DNAPTP6 condensation, Mark2‐rG4s can self‐organize into condensates to enhance DNAPTP6 LLPS [87]. A comparable mechanism is addressed in another study, which indicates that TERRA‐rG4s themselves undergo LLPS in a Ca^2+^‐dependent manner and act as nucleation centers (i.e., drivers that autonomously undergo phase separation) for α‐synuclein (α‐syn) LLPS, thereby promoting the creation of rG4s/α‐syn aggregates [31]. Notably, rG4 LLPS is necessary for rG4s/α‐syn condensate formation [31]. Furthermore, TERRA‐rG4s facilitate the in vitro LLPS of lysine‐specific demethylase 1 (LSD1) through direct binding [116]. However, the ability of TERRA‐rG4s to undergo LLPS was not investigated. Collectively, these findings suggest that the intrinsic LLPS capacity of G4s may be a critical, yet underappreciated variable in their interactions with diverse protein partners. Whether LSD1 condensation is driven by TERRA‐rG4s as nucleation centers or through a distinct mechanism warrants further investigation.

Overall, dG4s and rG4s both function as structural platforms to promote condensation, yet differ in their mechanistic roles. dG4s possess a dual capacity to both initiate and stimulate protein phase separation, whereas rG4s predominantly act as stimulators. Given the limited amount of research on G4‐regulated phase separation, these conclusions require further validation. Nonetheless, an appropriate molecular ratio of G4s to G4‐binding proteins is crucial for the effective assembly of condensates, illustrating the role of stoichiometry in defining condensate states. At relatively low concentrations, G4s cannot initiate LLPS, whereas higher concentrations of G4s dissolved the reconstituted condensates by interfering with the interaction between G4s and their binding proteins, as well as causing like‐like repulsive interactions (Figure 3B, bottom). Many studies have reported that higher concentrations of G4s can prevent condensation by G4‐binding proteins, as demonstrated by the examples of LSD1, HMGB1, and SP1 [104, 111, 116].

Beyond stoichiometry, the recruitment of client proteins further modulates condensate states. Rather than passive components [76], client proteins actively reinforce or weaken the G4‐protein network by participating in additional crosslinking or competing for binding interfaces. This client‐dependent regulatory mechanism explains why different proteins form condensates with distinct material properties when bound to the same G4 structure. This insight further implies that condensate states can be fine‐tuned by regulating the expression levels of client proteins. Therefore, stoichiometry and client recruitment may represent the key determinants of condensate state; this principle should be integrated into the predictive framework for G4‐mediated condensation.

Stoichiometry and client protein recruitment are extrinsic determinants, yet the capacity for G4‐regulated phase separation is also influenced by intrinsic structural features. The stability of G4 structures primarily depends on two critical factors: the number of G‐quartets and the length of loop regions. Generally, G4 structures with more G‐quartets and shorter loops exhibit higher stability [53]. However, the phase separation‐inducing capacity of G4s increases progressively with the number of G‐quartets and loop length [28, 90]. We hypothesize that structures containing more G‐quartets offer additional planar surfaces for multivalent π–π stacking, thereby enhancing multivalent interactions and reducing the saturation concentration required for phase separation. Notably, although longer loop regions destabilize G4s, they also provide additional protein‐binding interfaces and structural flexibility, which may facilitate phase separation. Thus, G4s possessing more G‐quartets and longer loop regions demonstrate superior phase‐separation promotion.

Having considered how G4s govern condensate formation and material properties, we next discuss their roles in regulating the phase transition between different material states.

### 3.3 G4s Regulate Phase Transitions

Phase transitions are tightly regulated by various factors that influence the strength or valency of intermolecular interactions, including protein concentration, post‐translational modification, composition, temperature, pH, and ionic strength [74, 117]. As discussed above, G4s are crucial structural platforms that decrease the phase‐separation threshold of G4‐binding proteins. Thus, G4s exhibit substantial potential for regulating the material properties of condensates and eventually inducing phase transitions (Figure 3C).

According to current literature on G4‐regulated protein phase separation, G4s primarily inhibit the dynamics of condensates, which manifest as a liquid‐to‐solid transition (Figure 3C, left). For example, the liquid‐to‐solid transition of FUS was markedly enhanced by interaction with rG4s; this regulatory effect was absent in FUS mutants deficient in G4 binding [112]. Following FUS binding to RNAs, its IDR may undergo conformational alterations, leading to an ordered state [118]. Therefore, the FUS phase transition induced by rG4s may be linked to the conversion of the disordered state of IDR into a stable form that drives these transitions [112]. Similarly, Asamitsu et al. demonstrated that condensates formed by the neurotoxic protein fragile X mental retardation 1 (FMR1) polyglycine (FMRpolyG) underwent a liquid‐to‐solid transition when exposed to CGG repeat‐derived rG4 (CGG‐rG4) [30]. During this transition, CGG‐rG4 initially forms hydrogel condensates, then binds to FMRpolyG within the droplets, facilitating the liquid‐to‐solid transition and FMRpolyG aggregation [30]. Thus, CGG‐rG4 may act as a nucleation center for the subsequent seed‐dependent aggregation of FMRpolyG.

Furthermore, Guo et al. used RNA oligonucleotides and cationic peptides (poly‐L‐lysine) as model systems to demonstrate that rG4‐containing oligonucleotides exhibit strong pairwise attraction to poly‐L‐lysine, leading to the formation of solid‐like states [119]. In contrast, the addition of less‐structured non‐G4 mutants induces poly‐L‐lysine to form liquid‐like droplets [119]. These findings suggest that rG4s promote the liquid‐to‐solid transition of poly‐L‐lysine. More recently, the Yabuki group reported that rG4s induce the liquid‐to‐solid phase transition in other G4‐binding proteins, including α‐syn and microtubule‐associated protein tau (Tau) [31, 120], and proposed the following mechanism by which rG4s induce α‐syn conformation and subsequent aggregation [31]. The α‐syn protein comprises three distinct domains: a positively charged N‐terminus, a hydrophobic non‐amyloid‐β component (NAC) domain, and a negatively charged C‐terminus [121]. Long‐range electrostatic interactions between the N‐ and C‐termini–along with hydrophobic interactions involving the C‐terminus and NAC region–contribute to a compact auto‐inhibitory conformation of α‐syn [31], which effectively limits exposure to NAC. However, the introduction of rG4s and Ca^2+^ may shield the N‐ and C‐termini, respectively, facilitating NAC exposure upon release of the compact conformation [31]. As a result, this mechanism leads to α‐syn adopting states prone to aggregation through interactions within the hydrophobic core domain (NAC‐NAC interaction).

Conversely, our group recently discovered that various types of dG4s promote MAZ transition from a gel‐like state to a liquid state in vitro (Figure 3C, right) [28]. MAZ possesses amino acid‐rich regions at both the N‐ and C‐termini along with six zinc finger (ZF) motifs in the central region [122]. The ZF3‐5 domains, rather than the disordered amino acid‐rich regions, are primarily responsible for MAZ phase separation [28]. Therefore, MAZ forms gel‐like condensates through close interactions between the ZF3‐5 domains in vitro. When dG4s bind to the ZF2‐4 domains, this interaction may partially weaken the connectivity between ZF3‐5 domains, enhancing the mobility of MAZ molecules within the condensates. However, this hypothesis warrants further investigation.

In summary, rG4s and dG4s play different regulatory roles in phase transitions. rG4s typically promote liquid‐to‐solid transitions, as demonstrated for FUS, FMRpolyG, α‐syn, and Tau. Notably, one study reported that rG4s only promote DNAPTP6 droplet formation but do not affect their dynamics [29], suggesting that not all rG4s drive liquid‐to‐solid transitions. In contrast, dG4s typically promote gel‐to‐liquid transitions, as demonstrated for MAZ.

The distinct outcomes of G4‐triggered phase transitions may not be solely dictated by dG4s or rG4s, but rather depend on whether G4s strengthen or weaken the dominant protein–protein interactions within the condensates. When G4s serve as structural platforms to stabilize exposed aggregation‐prone domains (e.g., by releasing the auto‐inhibitory conformation of α‐syn or templating FUS IDR ordering), they promote liquid‐to‐solid transitions. Conversely, when G4s compete with existing protein–protein interfaces (e.g., dG4s binding to MAZ ZF domains and weakening ZF–ZF interactions), they can fluidize condensates to promote gel‐to‐liquid transitions. This framework elucidates why most rG4s, primarily studied in the context of neurodegeneration, interact with aggregation‐prone proteins and drive solidification, whereas dG4s fluidize MAZ condensates by disrupting specific protein interfaces. This framework also explains how the same G4 can exhibit opposite effects depending on the specific protein partner and cellular context; however, this hypothesis warrants further verification.

Thus, G4‐triggered LLPS exhibits distinct patterns from liquid–solid transitions, which can be elucidated using a unified mechanistic framework. Specifically, liquid–solid transitions primarily manifest as stable, irreversible aggregates that are often closely associated with neurodegenerative diseases [30, 31, 67]. Mechanistically, this phenomenon is attributed to the strong multivalent interactions that drive rapid and irreversible fibril formation. In contrast, LLPS predominantly generates dynamic and reversible condensates that frequently perform various biological functions. The reversibility of these condensates arises from weak multivalent interactions, including π–π stacking between G‐quartets and electrostatic or hydrophobic forces. Such characteristics enable the dynamic modulation of condensates by cellular events such as transcription, stress, or viral infection [28, 29, 107, 113], thereby directly coupling phase separation to the spatiotemporal regulation of cellular functions. The biological roles of G4‐driven condensates are systematically summarized and discussed in Section 4.

## 4 Cellular Functions of G4‐Driven Condensates: From Physiology to Pathology

Following the mechanistic discussion in Section 3, this section describes the cellular functions of G4‐driven condensates. Depending on the cellular context, these condensates participate in normal physiological processes and contribute to various pathological conditions. Based on their functional outcomes (Table 1), we categorize condensates into two distinct types: physiological (Section 4.1) and pathological (Section 4.2).

### 4.1 G4‐Driven Physiological Condensates

**FIGURE 4:**
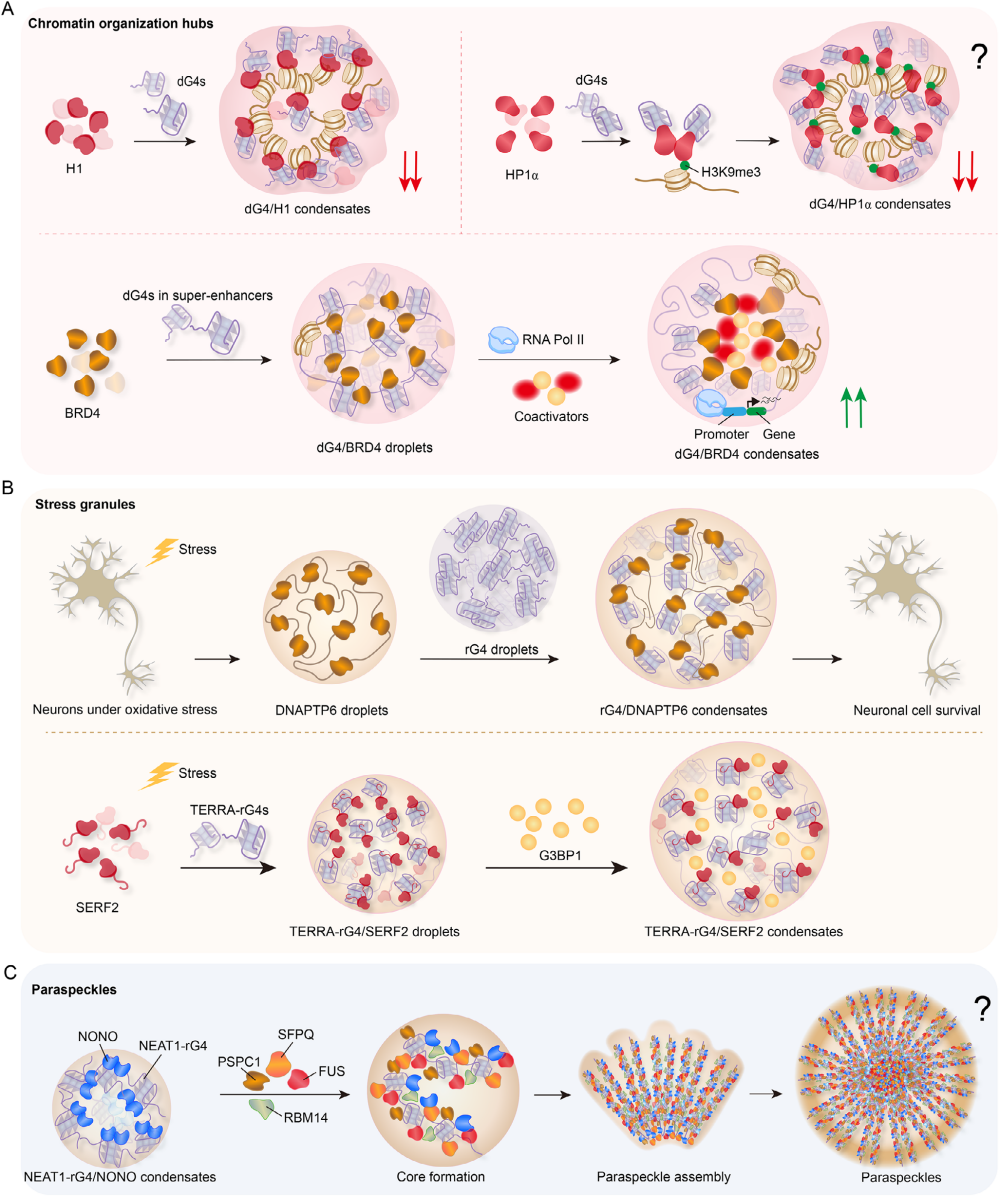
Mechanisms of G4‐driven condensation under physiological conditions (A) G4‐driven condensation as chromatin organization hubs. Based on in vitro data, DNA G‐quadruplexes (dG4s) promote the liquid–liquid phase separation (LLPS) of histone H1 through direct binding, leading to the formation of dG4/H1 condensates with poor molecular dynamics. These condensates may stabilize the nucleosome structure, resulting in heterochromatin formation and gene silencing (top left). Similarly, a hypothetical model is proposed for dG4/HP1α condensate formation: dG4s may directly bind to heterochromatin protein 1α (HP1α), leading to the formation of gel‐like dG4/HP1α condensates and heterochromatin (top right). At low concentrations, bromodomain‐containing protein 4 (BRD4) is unable to undergo LLPS. The super‐enhancer region forms G4s that facilitate the formation of BRD4 condensates, which further recruit transcriptional coactivators and RNA polymerase II (RNA Pol II) into these condensates, thereby forming super‐enhancers to activate gene transcription (bottom). The red and green arrows represent transcriptional repression and activation, respectively. (B) The assembly of stress granules via G4‐driven LLPS. Under stress conditions, many G4s and DNA polymerase‐transactivated protein 6 (DNAPTP6) are capable of undergoing LLPS, respectively. The interaction between RNA G‐quadruplex (rG4) and DNAPTP6 facilitates the formation of larger rG4/DNAPTP6 liquid condensates, which are crucial for the assembly of stress granules and the maintenance of normal neural cell survival (top). Supported by in vitro evidence, the condensate formed by telomeric repeat‐containing RNA G‐quadruplexes (TERRA‐rG4s) and small EDRK‐rich factor 2 (SERF2) interaction can further recruit Ras GTPase‐activating protein‐binding protein 1 (G3BP1) into their condensates, thereby promoting the assembly of stress granules (bottom). (C) Proposed model for rG4‐mediated paraspeckle formation (hypothetical). Specifically, the rG4s formed by nuclear paraspeckle assembly transcript 1 (NEAT1) bind to the non‐POU domain‐containing octamer‐binding protein (NONO), facilitating its phase separation to create NEAT1‐rG4/NONO condensates. These condensates subsequently recruit proteins essential for paraspeckle formation, such as splicing factor proline‐ and glutamine‐rich (SFPQ), paraspeckle component 1 (PSPC1), fused in sarcoma (FUS) protein, and RNA binding motif protein 14 (RBM14), thereby completing the assembly of paraspeckles through the phase separation mechanism. Note: Question marks (?) denote predicted mechanisms that lack experimental validation.

Membrane‐less organelles, such as SGs, P‐bodies, nucleoli, paraspeckles, and Cajal bodies, are essential for gene expression and protein synthesis under normal or stressed conditions [123]. LLPS governs the assembly and functionality of these organelles [25, 124, 125, 126]. As mentioned above, G4s facilitate the formation and phase transition of condensates through interactions with proteins. Consequently, phase separation mediated by G4 structural elements may represent a significant mechanism underlying membrane‐less organelle formation. This section presents examples of G4‐driven condensates, including chromatin organization hubs, SGs, and paraspeckles, and discusses their physiological roles in cells (Figure 4 and Table 1).

#### 4.1.1 Chromatin Organization Hubs

In the nucleus of eukaryotic cells, genomic DNA is highly compact and forms a nucleoprotein complex known as chromatin. The nucleosome, which serves as the basic unit of chromatin, comprises an octamer formed by two copies of each of the four core histones (H3, H4, H2A, and H2B) surrounded by 147 bp DNA. Linker DNA and histone H1 connect to the nucleosomes [127, 128]. The level of chromatin condensation reflects distinct chromatin states. Highly condensed chromatin (heterochromatin) inactivates gene transcription, whereas euchromatin, which is characterized by the lack of loosely packed nucleosomes, activates gene transcription [127]. LLPS governs chromatin organization and condensation by enhancing nucleosome packing. Gibson et al. found that reconstituted chromatin undergoes LLPS under physiological conditions, driven by the C‐terminal domain of H1 and the length of internucleosome linker DNA [129]. Additionally, Turner et al. showed that dsDNA promotes H1 LLPS, which helps to regulate chromatin organization by binding to internucleosome linker DNA [130]. In addition to dsDNA, dG4s promote the formation of H1 droplets in vitro (Figure 4A, top left) [82]. Notably, dG4s can reduce the molecular motility within these droplets, indicating possible inductive effects on heterochromatin formation, as heterochromatin behaves as a gel‐like state via phase separation [131], and the heterochromatin protein 1α (HP1α) can directly bind to G4s (Figure 4A, top) [132]. Although these in vitro findings suggest that dG4s function as hubs to modulate LLPS‐mediated chromatin condensation, whether the dG4‐driven condensation of H1 or HP1 is applicable *in cellulo* is unclear (Figure 4A, top). Therefore, current evidence suggests that dG4s contribute to heterochromatin organization without establishing their necessity or sufficiency *in cellulo*.

Despite previous reports on the repressive roles of G4s in gene transcription, substantial research indicates that G4s are primarily involved in gene activation [24, 133, 134, 135, 136, 137, 138]. LLPS occurs in certain euchromatin regions, including super‐enhancers, enhancer–promoter chromatin loops, and activated promoters [139, 140, 141]. Moreover, euchromatin regions are enriched with transcription co‐activators, such as BRD4, mediator complex subunit 1 (MED1), and active RNA polymerase II (RNA Pol II), which bind to G4s and form LLPS condensates [28, 140]. Therefore, G4s may enhance the recruitment of these factors, thereby maintaining an open chromatin state through LLPS. BRD4 plays a significant role in this mechanism. As a member of the bromodomain and extra‐terminal domain protein families, BRD4 is distinguished by its abundance in super‐enhancers and remarkable propensity for LLPS [140]. A recent analysis of whole‐genome data revealed that G4s are prevalent in human super‐enhancers, where they are excluded from the nucleosome while binding to BRD4 [107]. Instead of canonical right‐handed DNA, non‐canonical G4s located in super‐enhancers facilitate the formation of dG4/BRD4 condensates at physiological concentrations of BRD4, which are close to the dG4/BRD4 binding constant in vitro (Figure 4A, bottom). Consistently, the addition of G4 ligands significantly disrupts dG4/BRD4 condensates in vitro and inhibits genes regulated by G4‐rich super‐enhancers [107]. These findings suggest that dG4 folding within super‐enhancers is required to maintain BRD4 condensates and their transcriptional output, at least under conditions involving G4 ligand perturbation. However, no researchers have investigated whether dG4 folding alone is sufficient to drive condensate formation *in cellulo* in the absence of other super‐enhancer features, such as clustering of co‐activators including MED1 and cyclin‐dependent kinase 9 (CDK9).

Overall, the role of dG4‐driven phase separation in chromatin organization is context‐dependent. Current evidence supports a potential contributory role for dG4s in heterochromatin, whereas dG4 folding is required for full functionality in super‐enhancer‐associated euchromatin but is insufficient on its own.

#### 4.1.2 Stress Granules

When cells encounter various perturbations such as heat, oxygen radicals, ultraviolet light, nutrient depletion, and chemicals (e.g., arsenite), the intracellular protein translation system halts energy conservation and forms SGs that encapsulate mRNA, thereby protecting cells [123, 142]. SGs primarily comprise non‐translating mRNAs and RNA‐binding proteins, which govern diverse facets of RNA metabolism, including translation, sequestration of RNA‐binding proteins and mRNA molecules, and orchestration of signaling cascades [123, 143, 144]. In 2020, two independent studies indicated that SGs are cytoplasmic membrane‐less organelles formed through the LLPS of G3BP1 (a core SG protein) in conjunction with mRNAs [125, 145]. Notably, cytoplasmic rG4s have been linked to SG formation, as exemplified by rG4 enrichment within SGs under stress conditions [146, 147], enhanced SG formation through the transfection of oligonucleotides capable of forming rG4s [148], and the negative regulation of SG formation by the rG4‐helicases Bloom syndrome RecQ‐like helicase (BLM) and DEAH‐box helicase 36 (DHX36) [149, 150]. Furthermore, G3BP1 interacts with rG4s to contribute to SG assembly [115]. Thus, rG4s may be key structural components for SG formation.

Several studies have established that the rG4‐driven LLPS of RNA‐binding proteins, including DNAPTP6 and SERF2, contributes to SG assembly. Asamitsu et al. identified a novel rG4‐binding protein, DNAPTP6, in mouse neurons [29], whose knockdown disrupts SG formation under oxidative stress, leading to synaptic dysfunction and neuronal cell death. Mechanistically, DNAPTP6 promotes SG assembly in an rG4‐dependent manner, wherein rG4s undergo LLPS and enhance the LLPS of DNAPTP6 (Figure 4B, top) [29]. Their study elucidated valuable insights into the critical role of rG4‐dependent LLPS in SG assembly in normal neurons, but did not explore whether rG4 folding was necessary (e.g., by disrupting endogenous rG4s without globally altering RNA sequences) or sufficient (e.g., by inducing ectopic rG4 formation without cellular stress). Similarly, Sahoo et al. identified another rG4‐binding protein (SERF2) that facilitates SG formation. Importantly, TERRA‐rG4 promotes the formation of SERF2 droplets, which further facilitates G3BP1 condensation in vitro (Figure 4B, bottom) [108]. As described above, stress can lead to rG4 enrichment in SGs [147]. Under stress conditions, elevated rG4 levels may attract the assembly of a substantial amount of SERF2 protein into SGs (Figure 4B, bottom). Nevertheless, direct evidence of how rG4s regulate LLPS *in cellulo* is unavailable, and the necessity of rG4s for SERF2‐driven SG assembly under physiological stress has not been investigated.

In summary, rG4s formed in non‐translated mRNAs can recruit G4‐binding proteins and enhance their condensation, ultimately contributing to SG assembly. Current evidence does not support the notion that rG4 folding is necessary for SG formation under physiological conditions. Instead, rG4s may lower the threshold for SG assembly by providing multivalent structural interactions and by acting as a modulatory module that enhances condensate formation, rather than being an absolute determinant.

#### 4.1.3 Paraspeckles

Paraspeckles, or non‐membranous nuclear bodies, are subnuclear structures located in the interchromatin space adjacent to nuclear speckles [151, 152]. They are formed through associations between the long noncoding RNA nuclear paraspeckle assembly transcript 1 (NEAT1) and various RNA‐binding proteins, including non‐POU domain‐containing octamer‐binding protein (NONO), splicing factor proline‐ and glutamine‐rich (SFPQ), and paraspeckle component 1 (PSPC1) [123, 153, 154]. Paraspeckles are closely linked to several biological processes, including transcription, RNA transport, alternative splicing, and microRNA biogenesis [155, 156, 157, 158, 159]. The formation of paraspeckles is contingent on the LLPS of NEAT1 and its associated binding proteins. Yamazaki et al. revealed that NEAT1 serves as a structural platform for paraspeckle assembly by recruiting NONO dimers, which initiate the oligomerization of paraspeckle proteins, primarily SFPQ, PSPC1, FUS, and RNA‐binding motif protein 14 (RBM14). This process ultimately forms massive paraspeckle structures via LLPS [126].

Among the factors involved in paraspeckle assembly, NEAT1 is the most critical because of its essential structural role in LLPS initiation. However, the specific structural elements within NEAT1 that recruit NONO have not been identified. Simko et al. reported that rG4 structures in NEAT1 mediate these interactions [160]. Multiple rG4s are formed in NEAT1, and G4‐forming sequences are conserved across different species. Furthermore, NONO directly binds to NEAT1 rG4s with structural specificity, and the proteins that co‐immunoprecipitate with NEAT1 rG4s include SFPQ and PSPC1 (Figure 4C). Notably, treatment with 5,10,15,20‐Tetra(N‐methyl‐4‐pyridyl)porphine (TMPyP4), a G4‐binding compound, results in the dissociation of NONO‐NEAT1 complexes [160]. Thus, the rG4s located in NEAT1 may be necessary for the recruitment of NONO to NEAT1, a process that mediates the initial step of paraspeckle formation. (G4C2)n repeats‐derived rG4s promote the condensation of paraspeckle proteins, including NONO, SFPQ, and FUS; this activity is intensified by rG4 formation and diminished in the presence of TMPyP4 [148]. Furthermore, NONO, FUS, and RBM14 can independently undergo LLPS [161, 162, 163]. According to IUPred and VSL2 prediction algorithms, SFPQ and PSPC1 proteins also contain large IDRs, suggesting their potential to undergo LLPS. Therefore, rG4s present in NEAT1 may serve as a structural platform to initiate the LLPS of paraspeckle proteins and promote paraspeckle formation (Figure 4C). This compelling regulatory mechanism warrants further investigation.

### 4.2 G4‐Driven Pathological Condensates

**FIGURE 5:**
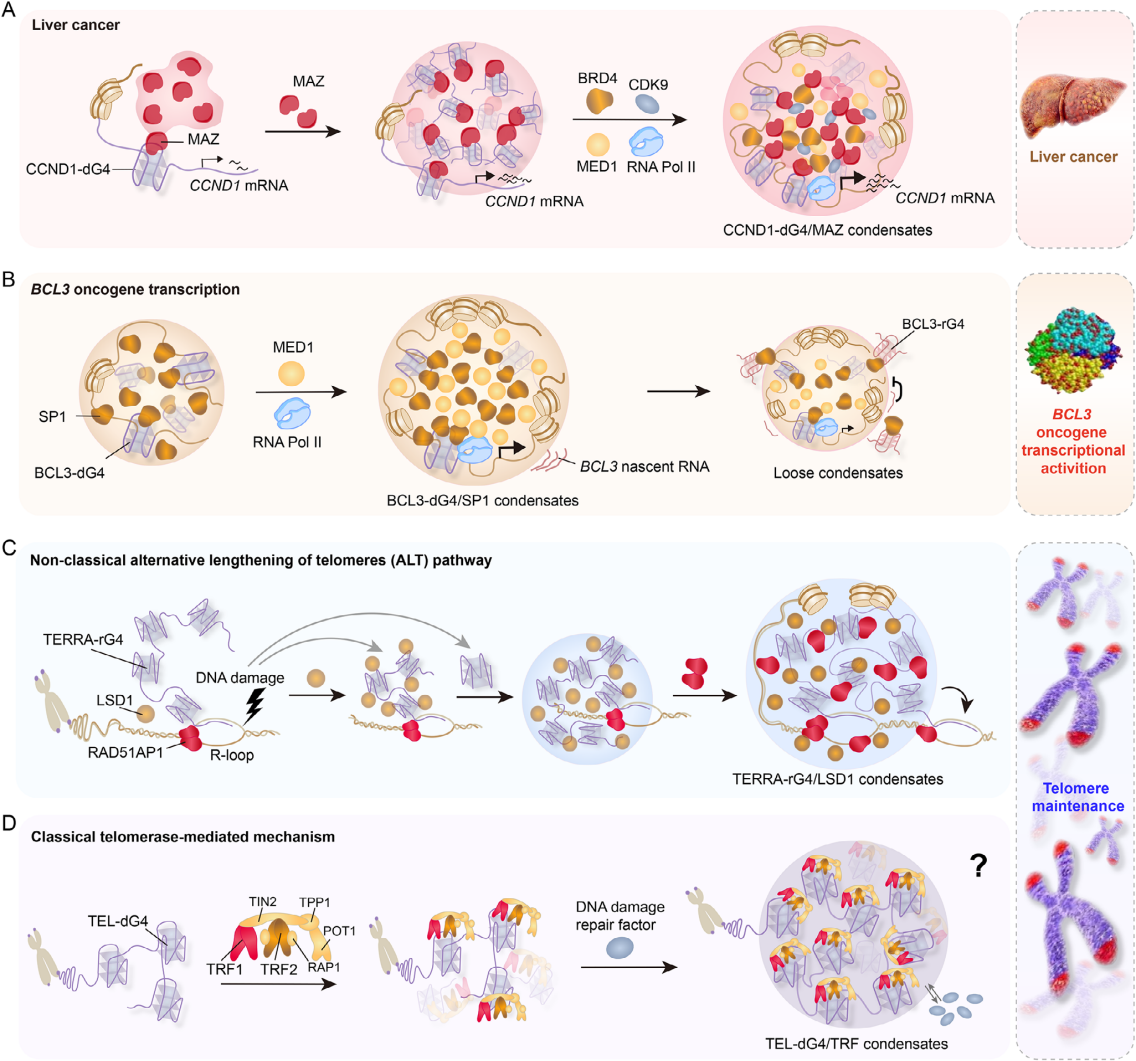
G4‐driven condensation in cancers. (A) The role of dG4 in promoting molecular motility of MYC‐associated zinc finger (MAZ) condensates to activate cyclin D1 (*CCND1*) gene expression. The dG4 structures formed by the *CCND1* promoter bind to MAZ and subsequently facilitate the (gel‐like)‐to‐liquid transition of its condensates. These liquid condensates can further recruit transcription activation‐associated factors, such as bromodomain‐containing protein 4 (BRD4), mediator complex subunit 1 (MED1), cyclin‐dependent kinase 9 (CDK9), and RNA polymerase II (RNA Pol II), resulting in the formation of CCND1‐dG4/MAZ condensates, which activate *CCND1* transcription and contribute to liver cancer development. (B) The role of dG4 in promoting the liquid–liquid phase separation (LLPS) of specificity protein 1 (SP1) to enhance B‐cell lymphoma 3 (*BCL3*) gene expression. Similar to the model in (A), the dG4 structures located in the *BCL3* promoter interact with SP1 and promote its LLPS. The process further attracts MED1 and RNA Pol II to assemble BCL3‐dG4/SP1 condensates, which activate *BCL3* transcription. Additionally, the BCL3‐dG4/SP1 condensates can be disrupted by the rG4s formed by the *BCL3* nascent RNA (BCL3‐rG4). (C) The role of telomeric repeat‐containing RNA G‐quadruplex (TERRA‐rG4) in maintaining telomere length. In the DNA damage‐mediated alternative lengthening of telomeres (ALT) pathway, a significant number of TERRA‐rG4s can bind to lysine‐specific demethylase 1 (LSD1), thereby inducing its LLPS. The subsequent recruitment of RAD51‐associated protein 1 (RAD51AP1) protein into TERRA‐rG4/LSD1 condensates facilitates R‐loop formation, which activates the ALT pathway for telomere maintenance. (D) A hypothetical model for telomere maintenance: telomeric dG4s promote telomerase phase separation. The dG4s located at telomeres may recruit telomerases, including telomeric repeat binding factor 1 (TRF1) and TRF2, along with other shelterin components, such as TRF1‐interacting nuclear factor 2 (TIN2), protection of telomeres 1 (POT1), and POT1‐TIN2 organizing protein (TPP1), to form liquid condensates. Furthermore, the TEL‐dG4/TRF condensates may selectively partition repressor/activator protein 1 (RAP1) while excluding DNA damage repair factors, potentially promoting telomere elongation by telomerases. Note: Question marks (?) denote predicted mechanisms that lack experimental validation.

**FIGURE 6:**
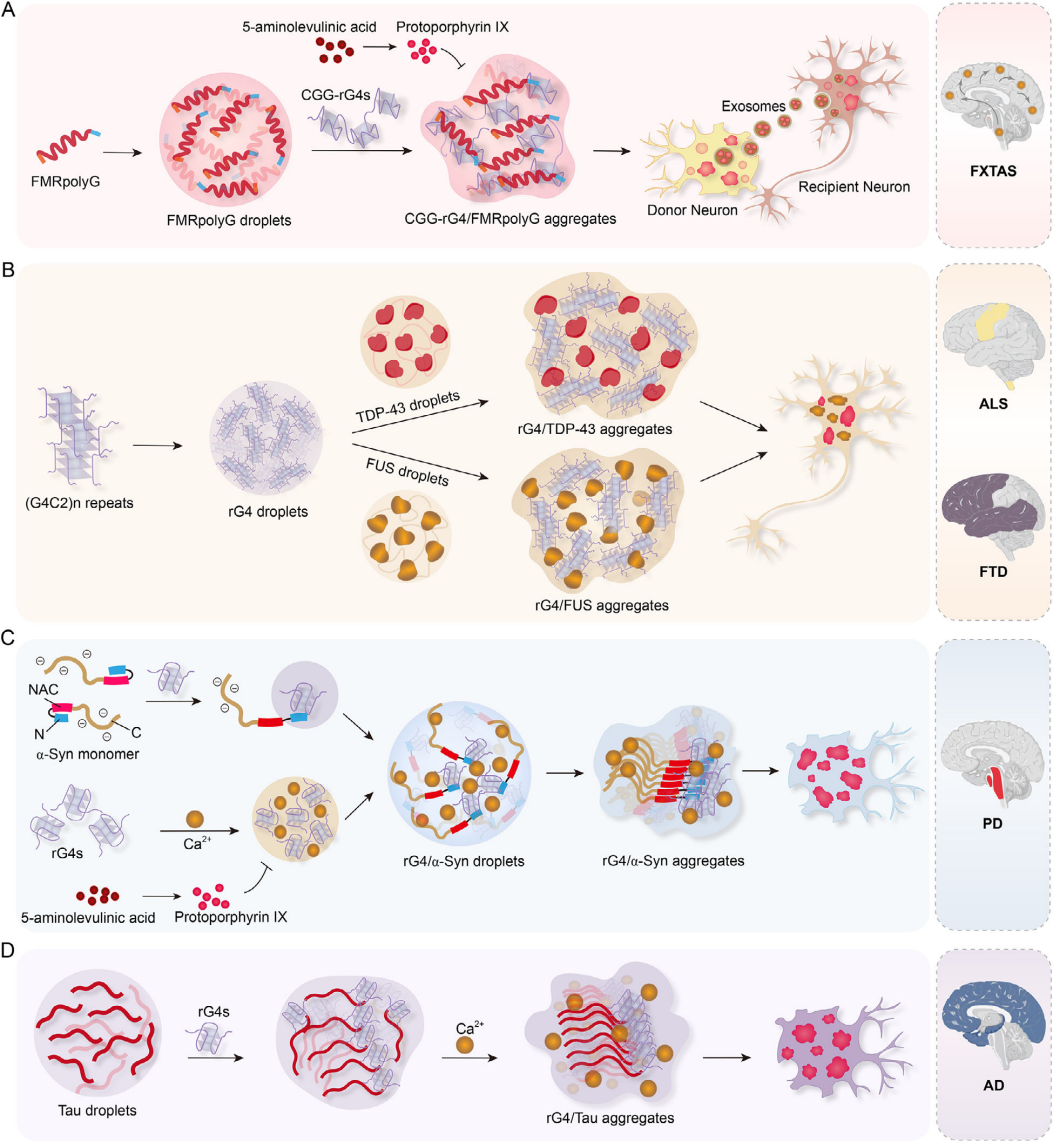
G4‐driven condensation in neurodegenerative diseases (A) The activity of RNA G‐quadruplex (rG4) to exacerbate neuronal damage through reducing molecular motility of fragile X mental retardation 1 (FMR1) polyglycine (FMRpolyG) condensates. FMRpolyG protein self‐assembles into liquid condensates. CGG repeat‐derived rG4s can bind to FMRpolyG and promote its liquid‐to‐solid transition to form CGG‐rG4/FMRpolyG aggregates. Notably, these aggregates can be transmitted from donor neurons to recipient neurons via exosomes, resulting in neuronal damage. 5‐aminolevulinic acid treatment can be metabolized to protoporphyrin IX, which directly binds to CGG‐rG4s, disrupts the condensates, and alleviates damage to nerve cells in fragile X‐related tremor/ataxia syndrome (FXTAS). (B) The potential role of (G4C2)n rG4s in amyotrophic lateral sclerosis (ALS) and frontotemporal dementia (FTD) development. From in vitro evidence, (G4C2)n repeats‐derived rG4s alone can form liquid condensates and promote the liquid‐to‐solid transition of transactive response DNA‐binding protein of 43 (TDP‐43) or fused in sarcoma (FUS) protein to form aggregates. These aggregates, potentially detrimental to neuronal cells, may contribute to the pathogenesis of ALS and FTD. (C) The role of Ca^2+^‐rG4 co‐condensation in Parkinson's disease (PD) development. Ca^2+^ can induce the self‐assembly of rG4s into liquid condensates. Furthermore, the rG4s and Ca^2+^ can bind to the N‐ and C‐terminal domains of α‐synuclein (α‐syn), respectively. These interactions promote α‐syn liquid–liquid phase separation (LLPS) and facilitate the formation of rG4/α‐syn aggregates through the exposure of the non‐amyloid‐β component (NAC) domain. The rG4/α‐syn aggregates are implicated in the pathogenesis of PD. The metabolite protoporphyrin IX produced by 5‐aminolevulinic acid disrupts the aggregates by directly binding to rG4 and ameliorates neuronal damage associated with PD. (D) The putative role of rG4s and Ca^2+^ cooperation in microtubule‐associated protein tau (Tau) aggregation and the acceleration of Alzheimer's disease (AD) pathogenesis. Similar to the model in (C), rG4s and Ca^2+^ also interact with Tau protein to promote the formation of rG4/Tau aggregates (based on in vitro data), which may contribute to AD development.

**FIGURE 7:**
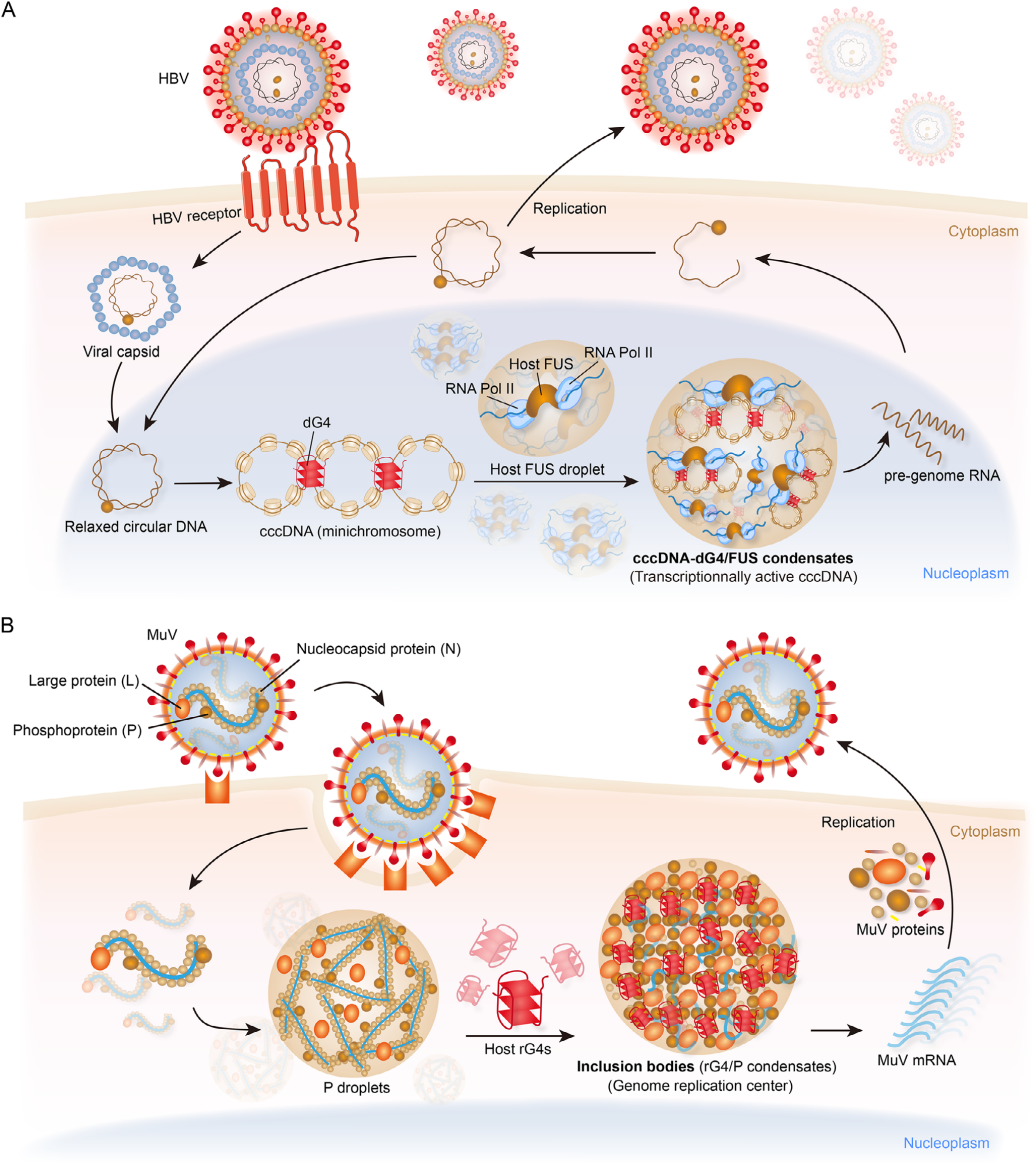
G4‐driven condensation in viral infection. (A) The function of dG4‐involved condensates to promote hepatitis B virus (HBV) production in infected hepatocytes. The HBV binds to receptors on the surface of hepatocytes, facilitating its entry into the cells and the subsequent release of genomic DNA after uncoating. Following this process, the relaxed circular DNA translocates into the nucleus, where it is converted into covalently closed circular DNA (cccDNA). The two G4 structures formed in the enhancer region of cccDNA interact with the host protein fused in sarcoma (FUS), which in turn recruits RNA polymerase II (RNA Pol II) to create cccDNA‐dG4/FUS condensates. These condensates are crucial for the activation of cccDNA transcription and the promotion of HBV replication. (B) Host rG4‐driven inclusion body formation and its potential role in mumps virus (MuV) infection. Upon entering the host cell, MuV releases its nucleocapsid into the cytoplasm. During RNA replication, the phosphoprotein (P) protein may interact with the nucleocapsid protein (N) and the RNA polymerase large (L) to form liquid condensates. As shown by in vitro experiments, the host rG4 structure can directly bind to the P protein and promote its liquid–liquid phase separation (LLPS), leading to the formation of rG4/P condensates. These condensates may encompass the N and L proteins along with viral RNA, forming inclusion bodies that may facilitate the replication of MuV viral RNA, thereby completing the MuV infection process.

Appropriate phase separation is crucial for maintaining the physiological functions of proteins. Consequently, aberrant phase separation may hinder the ability of proteins to perform normal functions, ultimately leading to disease onset. In this section, we introduce and discuss the roles of several abnormal condensates promoted by G4s in the context of disorders, including cancers, neurodegenerative diseases, and viral infections (Figures 5, 6, 7 and Table 1).

#### 4.2.1 Cancers

Cancer is associated with gene mutations or dysregulated gene transcription and is characterized by unrestricted cell proliferation [164]. Among the factors regulating gene expression, transcription factors play a pivotal role in coordinating these processes. Abnormal expression or mutations in transcription factors–such as tumor protein p53 and *MYC*–contribute to cancer development [165, 166]. Transcription factors activate gene transcription by directly binding to the promoters and/or enhancers of target genes [167, 168]. In this mechanism, transcription factors initially recognize specific binding sites on the genome (known as consensus motifs) and subsequently recruit transcriptional co‐activators (such as BRD4 and MED1) and transcription elongation complexes (including CDK7, CDK9, and RNA Pol II) to initiate and activate transcription [167, 169, 170]. In contrast to traditional transcription models, a non‐canonical model was recently proposed in which promoter G4s recruit transcription factors more efficiently than dsDNA to activate gene expression [99]. G4s act as oncogenic structural elements in cancers, as indicated by high G4 enrichment in cancerous tissues and cells compared to their normal counterparts [17, 22], as well as the presence of G4s in the promoters of numerous oncogenes [17, 45, 171]. However, the molecular mechanisms by which G4s recruit transcription factors and co‐activators to form transcriptional complexes that activate oncogene expression during cancer progression have not been elucidated. Recent advances in LLPS have provided a new framework for understanding the relationship between G4‐recruited transcription factors and cancer.

MAZ is a known G4‐binding transcription factor whose overexpression is implicated in the development of various solid tumors, including hepatocellular carcinoma, breast cancer, and pancreatic cancer [172, 173, 174, 175]. MAZ, which is increasingly expressed in liver cancer tissues and cells, can also form condensates [28]. Notably, the ability of MAZ to undergo condensation, both in vitro and *in cellulo*, depends on its capacity to bind to dG4s. For example, cyclin D1 (CCND1) promoter‐derived G4 (CCND1‐dG4) is essential for the formation of MAZ condensates exhibiting LLPS characteristics both in vitro and *in cellulo*, and was consequently named CCND1‐dG4/MAZ condensate. However, direct *in cellulo* evidence of the CCND1‐dG4 structure within MAZ condensates is lacking. Functionally, CCND1‐dG4/MAZ condensates serve as hubs that compartmentalize transcriptional co‐activators (such as BRD4 and MED1) and CDK9, thereby activating oncogenic *CCND1* expression and promoting cell proliferation and tumor growth in hepatocellular carcinoma (Figure 5A) [28]. However, whether this mechanism can be extended to all MAZ target genes or other cellular contexts remains unclear. Furthermore, although studies have shown that G4 binding is necessary for MAZ LLPS, none have shown whether G4 folding alone is sufficient to induce MAZ LLPS *in cellulo* without the presence of other factors, such as additional DNA elements and proteins.

Given that several transcription factors, including Yin Yang 1, SP1, early growth response 1, and CCCTC‐binding factor, possess the ability to bind G4s and undergo LLPS [141, 176, 177, 178, 179, 180, 181], the transcriptional activation mediated by G4‐driven MAZ LLPS may extend to additional transcription factors. Indeed, dG4s formed in *BCL3* promoter activate transcription by promoting SP1 LLPS and recruiting MED1 and RNA Pol II to form BCL3‐dG4/SP1 transcriptional condensates (Figure 5B) [111]. Moreover, rG4s formed on *BCL3* nascent RNA can compete with SP1 to bind to dG4s located in the *BCL3* promoter. This competition disrupts the structure of BCL3‐dG4/SP1 condensates, ultimately inhibiting *BCL3* transcription (Figure 5B) [111]. In the case of *BCL3*, the formation of SP1 condensates requires dG4 folding, whereas competing rG4s introduce a regulatory layer that tunes condensate stability. This further illustrates the contributory and context‐dependent nature of G4 functions.

In summary, researchers have shed light on how genomic G4s recruit transcription factors to activate gene transcription from the perspective of LLPS, further indicating that G4s primarily function as cis‐acting elements for transcriptional activation. For MAZ and SP1, G4 folding is necessary for condensate formation and transcriptional activation under the experimental conditions; however, G4 sufficiency has not been established. Therefore, G4s serve as necessary but insufficient cis‐acting elements in these oncogenic contexts. Future research should focus on designing anticancer drugs that disrupt G4s or hinder the binding of transcription factors to G4s, while acknowledging the possibility of variable G4 behaviors across different genomic loci and cellular states.

Replicative immortality is a crucial step in oncogenesis [164, 182]. Telomeres, which protect the chromosomal ends, play a central role in facilitating this process. Unlike normal cells, cancer cells maintain telomeric DNA at an adequate length to evade the onset of senescence or apoptosis. Telomere maintenance is evident in nearly all types of malignant cells and is primarily achieved by upregulating telomerase expression, which adds hexanucleotide repeats to telomeric DNA ends. Alternatively, the less common telomerase‐independent mechanism, known as the alternative lengthening of telomeres (ALT) pathway, utilizes homology‐directed repair to counteract replication stress‐induced telomere erosion [164, 182]. The transcription of TERRA from telomeres produced by RNA Pol II can be localized to other telomeres via hybridization with telomeric DNA to form R‐loops, a process that subsequently promotes DNA damage repair and activates the ALT pathway [183, 184, 185, 186, 187]. In addition to forming R‐loops, TERRA interacts with various proteins, including HP1, methyltransferase‐like 3, LSD1, and DNA repair factors such as FUS and breast cancer susceptibility gene 1, thereby facilitating telomere maintenance [185, 187, 188, 189, 190, 191, 192]. Recently, Xu et al. found that rG4s formed in TERRA can recruit LSD1 to DNA damage‐based ALT telomeres through direct interaction. This interaction may be enhanced by DNA damage, further enriching TERRA‐rG4s and leading to the formation of TERRA‐rG4/LSD1 condensates that specifically activate the ALT pathway by promoting R‐loop formation at telomeres [116]. Notably, the (R‐loops)‐stimulating protein RAD51‐associated protein 1 can partition into TERRA‐rG4/LSD1 condensates, thereby enhancing homology‐directed repair and telomere elongation (Figure 5C) [116]. This finding strongly supports the essential role of G4‐driven condensate formation in ALT pathway activation for telomere maintenance in cancer.

LLPS is linked to the classical telomerase‐mediated mechanism of telomere maintenance as well as the non‐classical ALT pathway. The Brangwynne group first revealed that repetitive telomeric DNA can function as a “super‐platform” that directly binds and drives the LLPS of telomerases containing telomeric repeat binding factor 1 (TRF1) and TRF2, subsequently promoting the formation of a liquid compartment with other key shelterin components, including TRF1‐interacting nuclear factor 2 (TIN2), protection of telomeres 1 (POT1), and POT1‐TIN2 organizing protein (TPP1) [193]. Shelterin condensates can selectively recruit the shelterin component, known as repressor/activator protein 1, and exclude access to DNA damage repair factors, preventing the degradation and fusion of chromosome ends [193]. Repetitive telomeric DNA can assemble into stable G4s, which play an essential role in telomere maintenance [194]. Moreover, telomerases, including TRF1 and TRF2, can bind G4s [195]. Thus, these distinctive G4 structural elements may mediate the platform role of telomeric DNA in driving the assembly of shelterin condensates (Figure 5D).

#### 4.2.2 Neurodegenerative Diseases

Neurodegenerative diseases include various disorders characterized by progressive degeneration and functional loss of neurons, such as Alzheimer's disease (AD), Parkinson's disease (PD), ALS, FTD, and Huntington's disease (HD) [196, 197]. Aberrant phase separation leads to protein aggregation, which is a key hallmark of these diseases [196, 198]. The transition from a reversible liquid state to an irreversible aggregate is predominantly regulated by disease‐associated mutations and post‐translational modifications [124, 162, 199, 200, 201]. Recently, the role of rG4s in promoting protein aggregation has been reported in neurodegenerative diseases. Here, we summarize examples of G4‐driven protein aggregation leading to neuronal damage for different disease types, including fragile X‐related tremor/ataxia syndrome (FXTAS), ALS/FTD, PD, and AD.

FXTAS is attributed to expansion of CGG triplet repeats within the 5′‐untranslated region of the *FMR1* gene [202, 203]. This expansion can result in repeat‐associated non‐AUG translation, leading to the production of a toxic polyglycine‐containing protein (FMRpolyG), which plays a crucial role in forming brain inclusions in individuals with FXTAS [202, 204]. As discussed in Section 3.3, rG4s formed in the CGG triplet repeats drive the liquid‐to‐solid transition and aggregate formation of FMRpolyG. Furthermore, CCG‐rG4/FMRpolyG aggregates propagate cell‐to‐cell through exosomes, leading to neuronal dysfunction (Figure 6A) [30]. Treatment with 5‐aminolevulinic acid, which is metabolized to protoporphyrin IX that binds to CGG‐rG4s, disrupts the formation of CGG‐rG4/FMRpolyG aggregates, ameliorating aberrant synaptic plasticity and behavioral deficits in FXTAS model mice (Figure 6A) [30]. Thus, CGG‐rG4s may serve as initiators of FXTAS pathogenesis by promoting FMRpolyG aggregation.

Similar to FXTAS, expansion of the hexanucleotide repeat (G4C2)_n_ found in the first intron of the *C9orf72* represents the most prevalent hereditary factor contributing to ALS/FTD [65, 66, 205]. The (G4C2)_n_ repeats form inter‐rG4s, which self‐assemble into condensates (Figure 6B). Transactive response DNA‐binding protein of 43 (TDP‐43) has been identified as a key causative protein of ALS/FTD [206, 207]. In vitro assays have shown that inter‐rG4s bind to TDP‐43 and promote the formation of pathological rG4/TDP‐43 aggregates [67], which are detrimental to neuronal cells (Figure 6B) [207]. In addition to TDP‐43, FUS aggregates are known pathogenic factors in ALS [208, 209]. In vitro data show that the liquid‐to‐solid transition and aggregation of FUS can also be caused by various types of G4s, including rG4s formed by (G4C2)_n_ repeats (Figure 6B) [112]. These findings indicate that the aggregate formation of ALS/FTD‐related proteins, including TDP‐43 and FUS, may be attributed to G4s formed by (G4C2)_n_ repeats. However, this conclusion is predominantly based on in vitro evidence and awaits further verification *in cellulo* and in vivo models of the disease.

PD belongs to synucleinopathies, primarily triggered by α‐syn aggregation [198, 210]. Additionally, continuous and excessive Ca^2+^ influx into neurons may strongly contribute to the onset of neurodegenerative disorders, such as PD and AD [211, 212]. Matsuo et al. demonstrated that the assembly of rG4 condensates induced by Ca^2+^ accelerates the phase transition and aggregation of α‐syn (Figure 6C) [31], as discussed in Sections 3.1 and 3.3. This mechanism and its role in nerve cell damage have been validated in both *in cellulo* and murine models. For example, in cultured mouse neurons treated with α‐syn preformed fibril, α‐syn co‐aggregated with rG4 condensates comprising synaptic mRNAs owing to an excess influx of cytoplasmic Ca^2+^, leading to neuronal dysfunction [31]. Moreover, the intentional induction of rG4 condensates via an optogenetic method triggered α‐syn aggregation, culminating in neuronal dysfunction and neurodegenerative effects. Notably, the oral delivery of 5‐aminolevulinic acid, which generates protoporphyrin IX to prevent the LLPS of rG4s, mitigated α‐syn aggregation, neurodegeneration, and motor‐defective progression in mice treated with α‐syn preformed fibril (Figure 6C) [31]. This finding suggests that Ca^2+^ influx‐induced rG4s condensates accelerate α‐syn aggregation, potentially contributing to PD. Similarly, Yabuki et al. identified both rG4s and Ca^2+^ as important factors inducing Tau aggregation (Figure 6D) [120], which is a hallmark of AD [213, 214]. Nonetheless, limited in vitro evidence highlights the need for further functional validation in both *in cellulo* and murine models. Additionally, the specific mechanisms by which rG4s and Ca^2+^ regulate Tau aggregation require investigation.

In summary, rG4s represent a novel factor driving the aggregation of proteins associated with neurodegenerative diseases, including FMRpolyG, TDP‐43, FUS, α‐syn, and Tau, which may advance neurodegeneration. Further research is needed to ascertain whether rG4s function as ubiquitous “master regulators” or as one of many parallel pathogenic pathways that converge upon protein aggregation.

#### 4.2.3 Viral Infection

Among infectious diseases, viral diseases pose the most significant threat to human health. As non‐cellular microorganisms, viruses rely on infected host cells to provide the essential raw materials, sites, energy, and other resources for virion replication and synthesis [215, 216]. LLPS plays a fundamental role in regulating viral life cycles through five key steps: viral adsorption; penetration and uncoating; genome replication and protein synthesis; new viral particle assembly; and particle release from infected host cells [217, 218, 219]. During viral infection, viruses enhance their reproductive efficiency by concentrating viral replication‐related biomolecules and/or intracellular components to form membrane‐less condensates, including viral replication compartments, cytoplasmic viral assembly compartments, and inclusion bodies [215, 220, 221, 222, 223]. Therefore, targeting condensates to inhibit viral replication represents a novel direction for antiviral drug development. Several research groups currently investigating the factors modulating condensate assembly have shown that G4s can induce condensate formation during viral infection.

The HBV genome comprises double‐stranded circular DNA with one strand remaining open, referred to as relaxed circular DNA [224, 225]. Upon hepatocyte infection, the viral genome is converted into cccDNA, which subsequently forms a true chromatin structure by associating with histones and other proteins within the host cells (Figure 7A). This chromatinized structure, known as the minichromosome, protects cccDNA and enables it to hijack the transcriptional machinery of the host cell, including RNA Pol II, to transcribe viral RNAs (Figure 7A) [226, 227, 228]. cccDNA formation and persistence in infected cells are key requirements for chronic HBV infections that prevent current treatments from fully eradicating the virus [229, 230]. Recently, Maadadi et al. reported that dG4‐induced transcriptionally activated phase‐separated compartments in infected hepatocytes are crucial for HBV replication [110]. Within cccDNA, two G4s form in the enhancer region. *In cellulo* studies have shown that cccDNA‐dG4s can recruit the host protein FUS and promote LLPS, thereby activating cccDNA transcription (Figure 7A) [110]. These findings reveal that HBV can hijack the physiological capacity of FUS to bind cccDNA‐dG4s and undergo LLPS, thereby benefiting from a nuclear environment that ensures optimal genome transcription. Therefore, cccDNA‐dG4s and FUS proteins in the host should be considered potential therapeutic targets in anti‐HBV drug development.

In addition to its role in transcriptional compartments, G4‐mediated LLPS plays a crucial role in forming inclusion bodies in virus‐infected cells; these are defined as nuclear or cytoplasmic aggregates of complete viruses and unassembled viral subunits, some of which comprise the reaction products of host cells in response to viral infection [217, 231]. In many cases, inclusion bodies are not merely passive aggregates, but organized structures exhibiting active viral replication and assembly [217, 232, 233]. For example, the synthesis of viral RNAs for mumps viruses (MuV) occurs within inclusion bodies [113]. MuV is the causative agent of mumps and poses a significant threat to children [234]. MuV particle cores predominantly comprise nucleocapsid proteins (N) that encapsulate viral RNAs, alongside RNA‐dependent RNA polymerases comprising large (L) protein catalytic subunits and phosphoprotein (P) cofactors (Figure 7B). Together, these three proteins constitute the viral ribonucleoprotein, which acts as the active center for genome replication [235]. Following MuV infection, inclusion bodies form in the cytoplasm of host cells via LLPS and are characterized by a cage‐like structure formed by N and P proteins, with viral polymerases distributed in a reticular pattern that co‐localizes with viral RNAs (Figure 7B) [113]. According to in vitro studies, abundant host rG4s within inclusion bodies interact with the P protein to promote condensation and form rG4/P condensates (Figure 7B) [113]. This suggests that host rG4s are crucial for concentrating the viral biomolecules required for effective viral RNA synthesis through LLPS and subsequently forming inclusion bodies during MuV infection. However, this model requires validation in the context of MuV‐infected cells.

## 5 Conclusions, Limitations, Challenges, and Future Perspectives

Genomic DNAs, transcription‐generated RNAs, and mature mRNAs assemble into a variety of non‐canonical nucleic acid secondary structures, including G4s, i‐motifs, and R‐loops. G4s are the most extensively studied structures, and their formation and unwinding are crucial for regulating normal cellular functions and disease onset. Unlike other structures, nucleic acid sequences can fold into various types of G4s, including intra‐G4s and inter‐G4s. Abundant recent research has confirmed that inter‐G4s can assemble *in cellulo*. Mechanistically, G4s can autonomously form condensates, act as structural platforms to initiate and stimulate condensate formation, or play an inductive role in phase transition. Functionally, G4‐driven condensates play significant roles in various physiological processes, including chromatin organization, SG formation, and paraspeckle assembly. However, aberrant condensates promoted by G4s are closely linked to the development of diseases such as cancer, neurodegenerative disorders, and viral infections. This review highlights G4s as emerging regulators of biomolecular condensation. In the following sections, we discuss several limitations of current validation methods and mechanistic studies in this field, identify key remaining challenges, and propose future research directions.

### 5.1 Methodological Limitations

#### 5.1.1 Limitation 1: Caveats of In Vitro and Cellular Evidence

A common challenge in the field of G4 condensation is that most mechanistic insights are derived from simplified in vitro reconstitution systems. While these systems have been instrumental in establishing fundamental principles, it remains largely unclear whether their conclusions can be directly extrapolated to the complex endogenous cellular environment. Most studies on G4‐related condensation have used in vitro reconstitution assays to explore the regulatory role of G4 structures in condensation (Table 1). These experiments provide significant advantages by eliminating interference from complex intracellular environmental factors, offering more direct evidence for condensation regulation through G4 and/or its binding proteins. However, in vitro reconstitution systems often rely on non‐physiological conditions, such as high concentrations of probes and proteins, crowding agents, and extreme ion concentrations. Endogenous cellular condensates are complex; thus, the resulting phase separation may not accurately reflect the true intracellular conditions.

Table S2 summarizes the in vitro reconstitution systems for G4‐driven condensation discussed in this review. Multiple studies have used non‐physiological concentrations of KCl and NaCl (e.g., too high or too low). In G4‐related experiments, excessively high KCl concentrations can significantly enhance G4 stability [12], potentially misrepresenting the intrinsic functions of G4s under physiological conditions. Notably, the types and concentrations of ions utilized vary significantly, generating inconsistencies regarding the influence of the same G4 structure on condensate properties. Furthermore, most studies utilize 10% PEG 8000 as a crowding agent to mimic crowded intracellular environments [28, 31, 104, 108, 111]. However, PEG 8000 can artificially induce or overstabilize condensates through nonspecific excluded volume effects [236]. This effect may generate false‐positive results and reduce molecular mobility within the condensates. Owing to these potential artifacts, conclusions regarding condensate dynamics from droplet fusion and fluorescence recovery after photobleaching (FRAP) assays require re‐evaluation. For the in vitro reconstitution of phase separation, we recommend using physiological ion concentrations and near‐physiological levels of nucleic acid probes and proteins. Orthogonal experiments with concentration gradients of ions, nucleic acids, and proteins are advised to determine critical thresholds for G4‐modulated condensation.

Similarly, *in cellulo* experiments, excessive exogenous probes, protein overexpression, and large fluorescent tags may interfere with the intrinsic properties of phase‐separated biomolecules [237, 238], making it difficult to link observations to endogenous scenarios. Additionally, some studies have explored G4‐mediated protein phase separation by constructing deletion mutants of the G4‐binding domain and analyzing subsequent condensate changes [28, 110, 116]. However, because the G4‐binding domain may interact with other nucleic acid motifs or proteins, this approach does not always determine the specific regulatory effects of G4 structures. Moreover, co‐localization of BG4 and G4‐interacting protein antibodies by immunofluorescence can support G4‐protein condensate formation. However, BG4 recognizes various G4 topologies and cannot distinguish between G4 structures derived from different sequences [18]. Therefore, more specific G4 probes should be developed that can recognize G4 structures with distinct topologies.

As a specific example of an alternative approach, a previous study on rG4s/α‐syn aggregates (discussed in Sections 3 and 4) adopted a novel “optoG4” system, which uses blue light to induce cryptochrome circadian regulator 2 (CRY2) oligomerization that subsequently promotes rG4 self‐assembly aggregation [31]. Although this system provided direct evidence of rG4‐induced α‐syn aggregation *in cellulo*, blue light stimulation is not an endogenous stress signal in neurological diseases, and its biological effects may therefore differ from those under actual pathological conditions. Additionally, blue light induction may elicit phototoxicity or oxidative stress *in cellulo*, indirectly affecting the G4 structure and phase separation [239]. Furthermore, the synergistic effects of tandem repeat sequences and CRY2 oligomerization may significantly drive local G4 density and aggregation beyond endogenous physiological thresholds [31]. Therefore, these findings warrant cautious interpretation. In addition to the optoG4 system, we recommend employing a CRISPR/Cas9 (Clustered Regularly Interspaced Short Palindromic Repeats/CRISPR‐associated protein 9) system to mutate endogenous G4‐forming sequences and disrupt G4 structures. This loss‐of‐function approach can help elucidate the role of G4 in phase separation.

#### 5.1.2 Limitation 2: Evidence From G4 Ligands and Probes

Beyond the methods discussed above, most current studies have indirectly investigated the regulatory role of G4s in phase separation through treatment with G4 ligands (Table S2). For example, G4 ligands have been introduced to in vitro or *in cellulo* contexts to evaluate the impact of G4s on the phase separation of their binding proteins. Although such experiments provide significant evidence for the role of G4s in condensation, several limitations are associated with G4 ligands and probes. In the context of G4‐regulated condensates, Table S2 lists commonly used G4 ligands and probes, including pyridostatin (PDS), TMPyP4, NMM, and SOP1812. However, each of these G4 ligands exhibits varying degrees of off‐target effects: (1) PDS can induce transcriptional pausing, DNA damage, topoisomerase inhibition, R‐loop accumulation, and replication stress [240, 241]; (2) TMPyP4 exhibits low specificity for G4 structures and may also bind dsDNA and i‐motif structures, potentially resulting in global alterations in gene expression [242, 243]; (3) the G4‐specific probe NMM can inhibit the activity of G4 helicases, such as BLM and RECQ [244]; (4) the next‐generation G4 ligand SOP1812 possesses enhanced specificity, but may still affect the expression of genes involved in various signaling pathways, including Wnt/β‐catenin and mitogen‐activated protein kinase [245]. Moreover, these G4 ligands or probes cannot discriminate between different G4 structures within cells, indicating insufficient specificity for targeting a particular G4. These off‐target effects indicate that G4 ligands may inadvertently alter condensate properties through nonspecific mechanisms, preventing the accurate analysis of specific G4 functions.

Furthermore, G4 ligand specificity is highly dependent on their concentrations. Different concentration ranges can exert distinct effects on condensation through various mechanisms, including dissolution or hardening. However, most studies do not evaluate concentration effects, leading to significant uncertainty when data are based on a single concentration. For example, the PDS may exhibit relative specificity at nanomolar concentrations (<1 µM), but its capacity to stabilize G4s is weak, resulting in minimal effects on condensates. Conversely, off‐target effects may arise in a concentration range of 1–10 µM, and the ligand primarily functions by specifically binding to G4s and inhibiting G4‐protein interactions, thereby disrupting condensate formation. At concentrations within 10–25 µM (the most common range, e.g., 20 µM), significant off‐target effects become nearly unavoidable [246, 247]. High ligand concentrations may dissolve liquid‐like condensates by disrupting their hydrophobic interactions or converting them into gel‐like states through off‐target crosslinking or oxidative damage. For example, 20 µM PDS can reduce dG4/BRD4 liquid‐like condensates and facilitate their transition from a liquid to gel‐like or solid‐like state in vitro [107]. The lack of systematic concentration titrations makes it challenging to ascertain the concentration range for differentiating between G4‐specific and nonspecific off‐target effects. Thus, the conclusion that G4 promotes condensation, predominantly achieved through G4 ligand experiments *in cellulo*, should be re‐evaluated to mitigate the risk of overinterpretation.

Thus, we recommend the following strategies for G4 ligand experiments: (1) conducting concentration gradient titrations and reporting the entire range tested, including concentrations without detectable effects; (2) employing multiple structurally unrelated ligands (if two or more ligands with distinct chemical structures yield similar effects, concerns regarding off‐target effects may be alleviated, although not entirely eliminated); and (3) implementing multiple orthogonal validation methods, such as mutating endogenous G4 sequences using CRISPR/Cas9 technology or performing helicase overexpression and knockdown experiments.

#### 5.1.3 Limitation 3: Methods for Identifying the Material Properties of Condensates

Current studies on G4‐regulated phase transitions primarily rely on FRAP to characterize the material properties of condensates (Table 1); however, this method has certain limitations. First, the interpretation of FRAP recovery curves can be ambiguous; incomplete or slow recovery does not necessarily indicate a solid or gel‐like state, as highly viscous liquids or viscoelastic fluids may exhibit similar characteristics [248, 249]. Conversely, rapid recovery may also arise from non‐liquid structures such as sponge‐like networks [250]. Second, FRAP results are significantly influenced by experimental parameters (e.g., bleach spot size and laser intensity), and variations in parameter settings across studies limit the comparability of quantitative results [248]. Even for the same condensate, reported FRAP recovery rates vary considerably. Thus, FRAP recovery kinetics do not provide clear criteria for classifying a condensate into liquid‐like or alternative phase states.

In addition to FRAP assays, some studies have relied on sensitivity to 1,6‐hexanediol as an indicator of LLPS. However, this compound primarily disrupts hydrophobic interactions but does not interfere with other mechanisms driving LLPS, such as electrostatic interactions, cation‐π interactions, π–π stacking, and specific molecular binding [251]. Furthermore, certain aggregates or fibrillar structures can be dissolved by 1,6‐hexanediol treatment [252]. Therefore, sensitivity to 1,6‐hexanediol cannot be considered a definitive criterion for confirming LLPS.

The methodological limitations described above, along with the inconsistent analytical criteria across studies discussed in Section 3 (e.g., differences in FRAP mobile fraction thresholds and morphological criteria), may lead to researchers classifying the same system into different material states or misinterpreting irreversible aggregation or gelation as LLPS. Future studies should therefore integrate multiple orthogonal methods for cross‐validation. Our specific recommendations are presented in Note S1.

### 5.2 Mechanistic Limitations

#### 5.2.1 Limitation 1: Reciprocal Effects of Condensates on G4 Stability

Direct intracellular evidence is limited to G4‐driven condensation, whereas in vitro experiments have provided substantial evidence that G4 provides a structural platform to promote condensation. However, the reciprocal effect of condensates on G4 structural stability has received little attention. Theoretically, the high local concentration and molecularly crowded microenvironment within condensates should favor G4 stabilization. Therefore, G4‐promoted condensation may further enhance its stability. This hypothesis has been supported by several studies. For example, in the (KRAS‐dG4)‐promoted HMGB1 phase separation discussed in Section 3.2, the resulting KRAS‐dG4/HMGB1 condensates stabilized KRAS‐dG4 structures [104]. Furthermore, disrupting phase separation with 1,6‐hexanediol caused global G4 destabilization in human chromatin [96], suggesting that phase separation helps maintain G4 stability.

However, some studies have shown that condensates confer helicase‐like activity on proteins and disrupt G4 structures. For example, the N‐terminal disordered tail of the DEAD‐box helicase 4‐truncated protein, which lacks a canonical helicase domain, can unfold G4 structures upon condensate formation [253]. Similarly, under non‐phase‐separating conditions, the FUS‐RGG domain only induces chemical shift perturbations of the imino protons in G‐quartet guanines when binding to TERRA‐rG4, with no disruption to the G4 structure. However, upon co‐condensation, more than one‐third of the TERRA‐rG4 structures can unfold, even in the presence of K^+^ [93].

This suggests a bidirectional regulatory mechanism between G4 structures and condensates. G4 structures can function as structural platforms that facilitate condensation, yet the unique microenvironment created by condensates can reciprocally influence the folding and stability of G4 structures by modulating local concentrations, molecular crowding, and interaction forces. Whether condensates promote or inhibit G4 folding likely depends on the properties of the involved binding proteins and the local cellular context, such as crowding, ionic environment, or the presence of other client proteins.

#### 5.2.2 Limitation 2: How Intracellular Factors Regulate G4‐Driven Condensation

Most current studies lack a systematic investigation of the influence of various environmental and cellular factors on the phase behavior of G4‐driven condensates. To date, few studies have reported that Ca^2^^+^ modulates the material state of G4‐driven condensates. For example, under normal conditions, TERRA‐rG4s can promote α‐syn to form liquid‐like condensates; however, under neurodegenerative disease conditions, significantly elevated intracellular Ca^2^^+^ levels can promote the transition of TERRA‐rG4/α‐syn condensates from liquid‐like to gel‐like or solid‐like states, ultimately leading to neuronal damage [31]. Intracellular K^+^ or Na^+^ play a crucial role in stabilizing G4 structures [12, 14, 34]; therefore, abnormal K^+^ or Na^+^ levels may regulate the phase behavior of G4‐driven condensates under pathological conditions such as cancer.

Another key class of regulators is G4 helicases. Helicases can maintain the dynamic turnover of G4 structures *in cellulo* [254]. Consequently, aberrant helicase expression may affect G4‐driven condensate formation and dissolution. However, few studies have reported a correlation between helicases and G4‐driven condensation. Under stress conditions, rG4 structure formation is significantly enhanced, leading to the recruitment of BLM helicases into SGs. Functional studies have demonstrated that BLM knockdown promotes SG formation, whereas exogenous BLM expression suppresses SG formation [149]. Similarly, exogenous DHX36 expression significantly reduced telomere clustering mediated by TERRA‐rG4/LSD1 condensates [116]. These findings suggest that helicases may disrupt the formation of SGs or telomeric condensates by unwinding rG4 structures. Conversely, low helicase expression levels may excessively enhance G4‐driven condensation, promoting the transition of condensates into stable solid‐like states. Therefore, maintaining appropriate expression levels of helicases is crucial for the dynamic regulation of G4‐driven condensation and for preserving normal cellular functions.

In addition to ions and helicases, client proteins may regulate G4‐driven condensation. However, most current studies have focused solely on G4 and its binding proteins, without testing the contributions of other client proteins within the condensates. Although client proteins may not undergo phase separation [76], they can interact with G4‐binding proteins and modulate their behavior in condensates. If client proteins possess enzymatic activities, they may promote the post‐translational modification of G4‐binding proteins and influence condensate properties. Therefore, identifying client proteins in G4‐driven condensates is crucial for understanding the mechanisms regulating condensate behavior.

Taken together, the phase behavior of G4‐driven condensates is dictated by the cellular environment. Typically, the same G4 structural platform can induce its binding proteins to form dynamic liquid‐like condensates under physiological conditions. However, pathological triggers, such as elevated ion concentrations, helicase dysfunction, or aberrant expression of client proteins, can induce a transition toward more stable, solid‐like aggregates. Thus, modulating these cellular factors may regulate the phase behavior of G4‐driven condensates and provide new therapeutic strategies.

### 5.3 Other Issues and Clarifications

#### 5.3.1 Issue 1: Why do G4s Promote Condensation More Than Other Nucleic Acid Structures?

Currently, few studies have examined the different roles of G4 and other secondary nucleic acid structures in regulating condensate formation, instead focusing on the molecular mechanisms by which specific nucleic acid structures interact with binding proteins to form condensates. We propose that G4s may possess a greater capacity to promote phase separation than other structures such as dsDNA, RNA, i‐motifs, and R‐loops. This predisposition can be attributed to three key features. First, the planar G‐quartet offers a large, polarizable aromatic surface that facilitates cation‐π and π–π stacking interactions [13, 42]. In contrast, dsDNA sequesters its bases within the helix, whereas RNA structures lack pre‐organized π‐rich surfaces. Second, the stacking of multiple G‐quartets results in a rigid, rod‐like architecture that enhances multivalent crosslinking along the long axis. Although dsDNA is rigid, it lacks regularly arrayed binding surfaces for multivalent interactions, whereas flexible RNAs rarely achieve pre‐organized global rigidity. Third, the variable loops and flanking sequences of G4 structures introduce intrinsic disorder and provide additional protein‐binding interfaces and structural flexibility; this reduces the entropy cost of condensation. The combination of a rigid G‐quartet core with flexibly disordered regions is uncommon in other nucleic acid structures. Specifically, dsDNA lacks intrinsic disorder; i‐motifs are pH‐dependent and do not possess stable, rigid π‐stacked cores at neutral pH [9]; and R‐loops are relatively transient and lack stable π‐rich surfaces [255].

In summary, the features of G4s lower the threshold for phase separation to an extent that is not achieved by other nucleic acid structures. Indeed, compared with dsDNA, CCND1‐dG4 significantly promotes the phase separation of MAZ protein and the molecular dynamics of condensates [28]. Future systematic comparative studies across diverse nucleic acid secondary structures are warranted to determine whether this G4‐favored phase separation mechanism can be extended to other G4‐forming loci and their cognate‐binding proteins.

#### 5.3.2 Issue 2: Comparing the Regulatory Roles of Intra‐ and Inter‐G4 Structures in Condensation

Current research on the regulation of protein condensation by inter‐G4 structures is limited. Therefore, we propose a prospective comparative study to address this gap. Specifically, intra‐G4 structures may function as dynamic conformational switches, with their folding and unfolding being highly dependent on cellular signals, such as changes in K^+^ concentration or helicase activity [14, 254]. This dynamic behavior allows for the reversible recruitment or release of client proteins, enabling the precise spatiotemporal regulation of dynamic condensates. In contrast, although inter‐G4s also respond to the aforementioned cellular signals, they tend to exhibit greater stability once formed. Inter‐G4s may act as stable multivalent structural elements that cross‐link separate and distant nucleic acid strands, resulting in condensates with reduced dynamics that subsequently recruit binding proteins to enhance structural stability and mechanical rigidity. However, only one study has demonstrated that inter‐G4s formed by (G4C2)n repeats promote the formation of pathological rG4/TDP‐43 aggregates, thereby contributing to the progression of ALS/FTD [67].

Based on these characteristic differences, we propose that intra‐G4s primarily participate in the formation of physiological and dynamic protein condensates, whereas inter‐G4s, if indeed involved in the phase separation process of proteins, are more likely to contribute to forming long‐term stable or pathological protein aggregates. To validate this hypothesis, new research tools must be developed to accurately distinguish and independently manipulate different G4 structures within complex intracellular environments.

### 5.4 Outstanding Questions and Experimental Strategies

In addition to the issues discussed above, we highlight several fundamental questions that remain unaddressed and propose experimental approaches to guide future research in this field.

**FIGURE 8:**
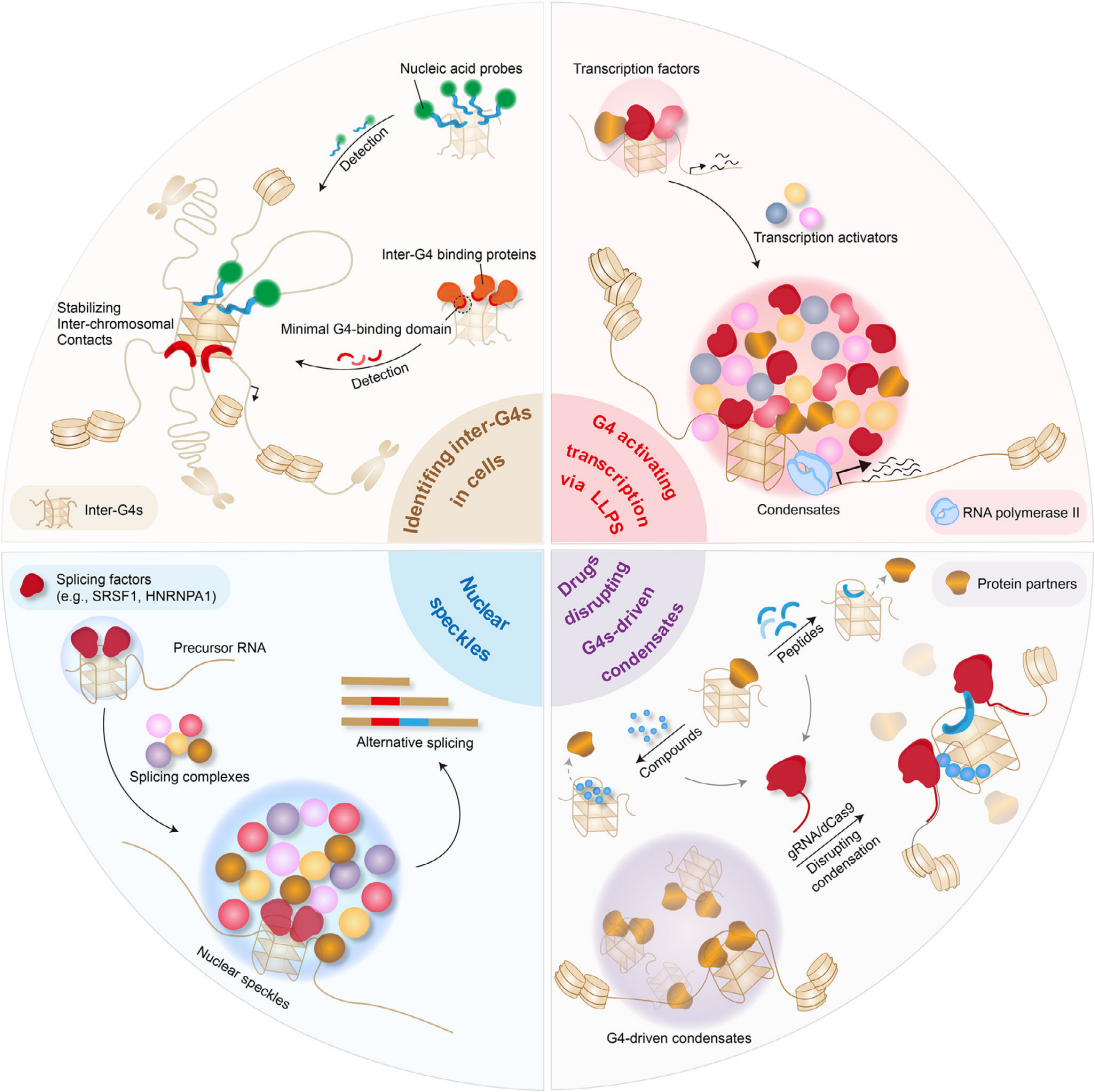
Future research projections in the field of G‐quadruplexes (G4s) and their regulated condensates (Top left) Different chromatin regions can be brought into proximity through protein complexes in bimolecular, trimolecular, and tetramolecular fashions, leading to the formation of intermolecular G4 (inter‐G4) structures. These inter‐G4s may be involved in stabilizing inter‐chromosomal contacts and orchestrating transcriptional programs. Screening nucleic acid probes or proteins that interact with inter‐G4s and designing polypeptide probes based on the minimal G4‐binding domain of proteins can facilitate the detection of inter‐G4s within living cells. (Top right) Promoter G4s can recruit transcription factors and coactivators to form condensates for transcriptional gene activation. (Bottom left) RNA G‐quadruplexes (rG4s) formed near splice sites of precursor RNA can bind splicing factors, such as serine/arginine‐rich splicing factor 1 (SRSF1) and heterogeneous nuclear ribonucleoprotein A1 (HNRNPA1), to promote their phase separation. These condensates further recruit additional splicing complexes to assemble condensates, thereby facilitating alternative splicing and nuclear speckle formation. (Bottom right) Small‐molecule compounds and peptides that specifically bind to G4s can be screened and identified. These compounds or peptides can be conjugated to nuclease‐dead Cas9 (dCas9) and targeted to a specific G4 structure using guide RNA (gRNA). This strategy effectively inhibits the interaction between the G4 and its protein partners, thereby disrupting the condensate.

First, inter‐G4 structures should be identified within cells using both nucleic acid‐based probes and peptide‐based tools (Figure 8, top left). This can be accomplished through (1) structure‐guided selection methods–such as the systematic evolution of ligands by exponential enrichment–to develop nucleic acid probes that are specific for inter‐G4s; (2) proximity labeling techniques–such as engineered ascorbate peroxidase 2 or proximity‐dependent biotin identification–in conjunction with mass spectrometry to objectively identify inter‐G4‐binding proteins. This should be followed by peptide design based on the minimal G4‐binding domain of identified proteins and their conjugation with fluorescent tags for validation; (3) orthogonal validation using multiple probes–such as the BG4 antibody and small‐molecule ligands–to further confirm probe specificity. We predict that inter‐G4s provide the structural basis for interactions between different chromosomes, potentially stabilizing inter‐chromosomal contacts and orchestrating transcriptional programs (Figure 8, top left).

Second, future research should explore the universality of the LLPS mechanism by which G4s recruit transcription factors to activate gene transcription. Transcription factors, such as MAZ and SP1, exert transcriptional activation functions via the aforementioned mechanisms [28, 111]. We speculate that additional transcription factors capable of binding to G4s and undergoing LLPS may also recruit transcriptional activators to condensates via the G4‐mediated LLPS mechanism, thereby efficiently facilitating transcription (Figure 8, top right). To test this hypothesis, future studies should (1) investigate G4‐driven transcriptional condensate formation *in cellulo* using orthogonal live‐cell validation with multiple probes; (2) perform endogenous knock‐in of G4‐disrupting synonymous mutations in the promoter regions of candidate genes to establish causality; (3) conduct quantitative phase‐diagram mapping to define the biophysical principles governing G4‐driven transcriptional condensates; and (4) measure the transcriptional activation of G4/transcription factor condensates using nascent RNA fluorescence in situ hybridization.

Third, the role of G4‐regulated LLPS mechanisms in the formation of nuclear speckles warrants further investigation. Nuclear speckles are membrane‐less organelles formed through LLPS that serve as crucial sites for the alternative splicing of precursor RNAs [123]. G4s form on precursor RNAs and can regulate alternative splicing [256]. Therefore, we hypothesize that G4s function as structural platforms, recruiting splicing factors such as serine/arginine‐rich splicing factor 1 and heterogeneous nuclear ribonucleoprotein A1 to efficiently assemble splicing complexes through LLPS (Figure 8, bottom left). This hypothesis should be validated through the following approaches: (1) mapping G4 positions on pre‐mRNAs enriched in nuclear speckles using G4‐specific RNA immunoprecipitation sequencing; (2) conducting orthogonal live‐cell validation with multiple probes to confirm the colocalization of G4s and splicing factors within nuclear speckles; (3) quantitatively mapping phase‐diagrams to determine how G4 concentration and RNA sequence affect splicing condensate formation; (4) performing endogenous knock‐in of G4‐disrupting synonymous mutations in pre‐mRNA G4 sequences to evaluate the necessity of G4 folding for splicing condensate formation and alternative splicing; and (5) assessing the generality of G4‐driven LLPS in modulating alternative splicing through RNA‐sequencing.

Finally, future research should focus on screening compounds or designing peptides that can disrupt condensates driven by G4s. Because condensate formation depends on interactions between G4s and proteins, we hypothesize that compounds or peptides capable of directly interfering with specific G4‐protein interactions may effectively inhibit the formation of these condensates. The G4‐binding compounds of interest primarily include hybrid polycyclic aromatic molecules, macrocyclic molecules, and modular aromatic structures [257], such as the previously reported protoporphyrin IX [30, 31]. Furthermore, the peptide design can be optimized based on the minimal domains within the proteins that facilitate G4 binding (Figure 8, bottom right). Given that these compounds or peptides may not specifically bind to various G4s, off‐target effects remain a significant concern. To address this issue, we have adopted a strategy recently established by Qin et al. [258]. They experimentally demonstrated that conjugating G4‐stabilizing proteins or ligands to a nuclease‐dead Cas9 (dCas9) enables selective stabilization of G4s at specific genomic loci through sgRNA guidance, without globally perturbing other endogenous G4s [258]. This provides a validated molecular tool for spatially precise G4 targeting.

Building on this design principle, we extrapolate that the screening of compounds or peptides could, by extension, be coupled with dCas9. This adaptation may offer a pathway to inhibit G4‐driven condensation of interest with enhanced spatial selectivity. However, it is important to emphasize that this strategy remains entirely hypothetical at this stage, and its therapeutic potential awaits rigorous experimental validation. Nevertheless, we anticipate that the integration of dCas9‐based targeting with high‐throughput compound screening may provide promising avenues for specifically dissolving G4‐driven pathological condensation, a direction that warrants systematic investigation in future studies.

## Author Contributions

W.W., G.S., and D.L. conceived and authored the manuscript. W.W. and D.L. were responsible for creating the figures. W.W., D.L., Q.X., and Y.Z. conducted literature searches and organized the findings. F.L. contributed conceptual suggestions. All authors have read and approved the final version of the manuscript for publication.

## Conflicts of Interest

The authors declare no conflicts of interest.

## Supporting information




**Supporting File**: advs76663‐sup‐0001‐SuppMat.docx.

## Data Availability

Data sharing not applicable to this article as no datasets were generated or analysed during the current study.
