# Supplementary material for: G‐Quadruplexes: Structural Diversity and Emerging Roles in Biomolecular Condensation
Source: Adv Sci (Weinh). 2026 Jul 20:e76663. Online ahead of print. doi: 10.1002/advs.76663 (PMC13383166; doi:10.1002/advs.76663)
Supplement: Supplementary file 1 — Supporting File: advs76663‐sup‐0001‐SuppMat.docx. [file ADVS-9999-e76663-s001.docx]

**Supplementary** **Materials**

G-quadruplexes: Structural Diversity and Emerging Roles in Biomolecular Condensation

Wenmeng Wang^a, 1^, Qingqing Xu^a,1^, Yuxin Zhang^a^, Fang Liu^b^, Guangchao Sui^a,^*, Dangdang Li^a,^*

^a^ College of Life Science, Northeast Forestry University, Harbin 150040, China.

^b^ Department of Medical Oncology, Harbin Medical University Cancer Hospital, Harbin 150081, China.

* Correspondence: gcsui@nefu.edu.cn; lidd@nefu.edu.cn; Tel: +86-451-82191081

(^1^These authors contributed equally to this work.)

**Supplementary Note 1. Common experimental metrics for classifying the material states of biomolecular condensates**

Characterization of the material properties of biomolecular condensates is essential for understanding their biological functions. Multiple methods have been developed to distinguish between the liquid-, gel-, and solid-like states of condensates. Here, we summarize the common experimental metrics for assessing condensate material properties, which are categorized into interfacial properties, molecular dynamics, and rheology (Table S1). The criteria in Table S1 are commonly used indicators rather than absolute standards, as material properties exist along a continuum and may vary with experimental conditions.

(1) Interfacial properties describe interactions between the condensate surface and surrounding environment, which is primarily assessed through surface tension. Confocal microscopy offers a direct method for observing the fusion behavior of condensates. Surface tension determines the sphericity and fusion efficiency of condensates; lower surface tension facilitates a spherical shape and rapid fusion, whereas higher tension leads to irregular shapes and diminished fusion efficiency [S1, S2].

(2) Molecular dynamics elucidates the dynamics of molecules within condensates and their exchange rates with the surrounding environment. Fluorescence recovery after photobleaching (FRAP) is the most common method for evaluating molecular dynamics, as it records the fluorescence recovery process in a bleached region, enabling the calculation of recovery fractions and half-times (FRAP recovery fraction and t₁/₂) [S3, S4]. Fluorescence correlation spectroscopy (FCS) is a high-precision technique that measures diffusion coefficients by analyzing the Brownian motion of molecules [S5]. Other validation methods included fluorescence loss in photobleaching (FLIP) and pulsed-field gradient nuclear magnetic resonance (PFG-NMR) [S4, S6].

Furthermore, physicochemical perturbations, such as concentration, salt, temperature, pH, and condensate-disrupting chemicals (e.g., 1,6-hexanediol), provide orthogonal validation of the material states of condensates.

(3) Rheology describes the overall mechanical response of condensates to external forces or deformation, which is primarily assessed by viscoelasticity. *In vitro*, condensate viscoelasticity can be measured using techniques such as optical tweezers, atomic force microscopy (AFM), or particle-tracking micro-rheology, which quantify the mean-squared displacement (MSD) of nanoparticles [S2]. Additionally, condensate viscosity can be indirectly assessed through fusion assays, as described in Section (1).

**Supplementary Table S1. Criteria for distinguishing liquid-, gel-, and solid-like condensates**

| **Category** | **Property** | **Liquid-like** | **Gel-like** | **Solid-like** | **Method** |
| --- | --- | --- | --- | --- | --- |
| Interfacial property | Surface tension | Low (rapid fusion, spherical) | Moderate (slow fusion, irregular) | High (no fusion, fibrillar) | Confocal microscopy (fusion assay) |
| Molecular dynamics | Molecular mobility | Fast FRAP recovery (high mobile fraction) | Slow or partial FRAP recovery | No FRAP recovery | FRAP |
|  | Diffusion | Brownian motion | Anomalous sub-diffusion | No detectable diffusion | FCS |
|  | Environmental responsiveness | Reversible, saturated concentration-dependent, 1,6-hexanediol-sensitive (if hydrophobically driven) | Partially reversible or irreversible, may resist 1,6-hexanediol | Irreversible, resistant to 1,6-hexanediol | Dilution, temperature shift, 1,6-hexanediol |
| Rheology | Viscoelasticity (macroscopic) | Viscous (flows, recovers rapidly) | Elastic (recoils, may fracture) | Rigid (no deformation) | Optical tweezers, AFM |
|  | Viscoelasticity  (microscopic) | Free diffusion (high MSD) | Sub-diffusion (reduced MSD) | No motion | Particle-tracking micro-rheology |
|  | Apparent viscosity | Low viscosity (rapid fusion) | Moderate viscosity (slow fusion) | High viscosity (no fusion) | Confocal microscopy (fusion assay) |

Footnotes: The criteria outlined above should be interpreted as commonly used reference indicators rather than universally applicable thresholds. Liquid-, gel-, and solid-like states often exist along a continuum, and reported values (e.g., FRAP recovery half-times) may vary depending on experimental conditions, including protein concentration, buffer composition, and temperature. These criteria should be evaluated in conjunction with orthogonal assays and within the specific context of each experimental system.

(4) Recommended orthogonal assays. No single assay is suitable for this purpose; therefore, FRAP, fusion, and morphological assessments should be combined. Where available, techniques such as FCS, optical tweezers, AFM, and particle-tracking micro-rheology are recommended to further quantify the material properties.

**Supplementary Table S2. Experimental conditions for *in vitro* and *in cellulo* (or *in vivo*) evidence of G4-driven condensation (corresponding to Table 1)**

| **Condensate** | **Key evidence *in vitro*** | **Key evidence** ***in cellulo* (or *in vivo*)** | **Ref.** |  |
| --- | --- | --- | --- | --- |
| *C9orf72* r(G4C2) G4 alone | **Ionic:** 10 mM MgCl_2,_ 25 mM NaCl, 10 mM Tris-HCl buffer (pH 7.5)  **Other:** 100 mM NH_4_OAc; 1 mM doxorubicin (disrupt rG4 condensates) | U2OS cells, overexpression of r(G4C2)_29_  **Other:** 100 mM NH_4_OAc; 2.5 μM doxorubicin (disrupt rG4 foci) | 87 |  |
|  | **Ionic:** 500 mM KCl, 10 mM Tris-HCl buffer (pH 7.4)  **Crowding agent:** 30% PEG 200  **G4 ligand:** 10 μM PDS (disrupt rG4 condensates) | Induced pluripotent stem cell (iPSC)-derived motor neurons, overexpression of (GGGGCC)_9_ oligos | 67 |  |
| Small RNAs (e.g., GCGGCGGC, CCGGGGCC) G4 alone | **Ionic:** 150 mM KCl, 25 mM MgCl_2_, 10 mM Tris-HCl buffer (pH 7.5)  **Crowding agent:** 10%-40% PEG 200  **Other:** 0.5 mM complementary ASO; 2 μM doxorubicin; 10% 1,6-hexanediol (disrupt rG4 condensates) | HeLa cells, overexpression of rG4;  Xenograft tumor, injection of rG4-forming RNAs  **Other:** 2 μM doxorubicin; 10% 1,6-hexanediol (no response) | 86 |  |
| Poly(UG) G4 alone | **Ionic:** 150 mM KCl, 20 mM Tris-HCl buffer (pH 7.0)  **Crowding agent:** 10% PEG 8000 | No experimental evidence | 84 |  |
| SHR mRNA G4 alone | **Ionic:** 100 mM KCl, 10 mM Tris-HCl buffer (pH 7.5)  **Crowding agent:** 10% PEG 8000 | plant root cells, overexpression of SHR RNAs | 90 |  |
| *Mark2* mRNA G4 alone | **Ionic:** 0-250 mM MgCl_2_, 25 mM NaCl, 10 mM Tris-HCl buffer (pH 7.0) | No experimental evidence | 29 |  |
| TERRA-rG4 alone | **Ionic:** 140 mM KCl, 10 mM MgCl_2_, 150 mM NaCl, 2 mM CaCl_2,_ 50 mM Tris-HCl buffer (pH 7.5)  **G4 ligand:** 10 μM PPIX (disrupt rG4 condensates)  **Other:** 2 mM EGTA (disrupt rG4 condensates) | No experimental evidence | 31 |  |
| dG4/H1 | **Ionic:** 0-300 mM NaCl, 10 mM Tris-EDTA buffer (pH 7.4), at room temperature  **Crowding agent:** 15% PEG 4000  **Other:** 20% 1,6-hexanediol (disrupt dG4 condensates) | No experimental evidence | 82 |  |
| dG4/HMGB1 | **Ionic:** 20 mM KCl, 10 mM HEPES buffer (pH 7.4)  **Crowding agent:** 10% PEG 8000 | No experimental evidence | 104 |  |
| dG4/RECQ4 | **Ionic:** 70 mM KCl, 25 mM KPO_4_ (pH 6.5), at room temperature | No experimental evidence | 105 |  |
| dG4/ZNF706 | **Ionic:** 20 mM KCl, 20 mM NaPi buffer (pH 7.4), at room temperature | HEK-293T cells, endogenous G4 + endogenous ZNF706 | 106 |  |
| dG4/BRD4 | **Ionic:**125 mM NaCl, 10 mM Tris–HCl buffer (pH 8.0), at room temperature  **Crowding agent:** 20% PEG 400  **G4 ligand:** 20 μM PDS; 1.25 μM SOP1812 (liquid-to-solid transition)  **Other:** 10% 1,6-hexanediol (disrupt dG4/BRD4 condensates) | No experimental evidence | 107 |  |
| dG4/SERBP1 | No experimental evidence | Giant membrane vesicles derived from HeLa or HepG2 cells, overexpression of (G3A)_4_-G4 + endogenous SERBP1 | 109 |  |
| cccDNA-dG4/FUS | No experimental evidence | HBV-infected HepG2 cells, overexpression of FUS wild type (wt) or mutants | 110 |  |
| BCL3-dG4/SP1 | **Ionic:** 100 mM KCl, 10 mM MgCl_2_, 10 mM HEPES buffer (pH 7.3)  **Crowding agent:** 10% PEG 8000 | MDA-MB-231 cells, endogenous SP1 and G4  **G4 ligand:** 10 or 20 μM TMPyP4 (disrupt SP1 puncta) | 111 |  |
| rG4/SERF2 | **Ionic:** 100 mM KCl, 20 mM NaPi buffer (pH 7.4), at room temperature  **Crowding agent:** 10% PEG 8000 | U2OS cells, endogenous SERF2 and G4, under stress conditions | 108 |  |
| rG4/P | **Ionic:** 150 mM NaCl, 20 mM Tris-HCl buffer (pH 7.5), at room temperature  **Crowding agent:** 5% PEG 8000 | No experimental evidence | 113 |  |
| rG4/APE1 | **Ionic:** 50 mM KCl, 10 mM MgCl_2_, 50 mM Tris-HCl buffer (pH 7.5) | No experimental evidence | 114 |  |
| Mark2-rG4/DNAPTP6 | **Ionic:** 150 mM NaCl, 10 mM Tris-HCl buffer (pH 7.5), at room temperature | Neuro-2A cells, overexpression of Mark2-rG4 + endogenous DNAPTP6  **Other:** 0.5 mM NaAsO2 (stress condition) | 29 |  |
| TERRA-rG4/LSD1 | **Ionic:**100 mM NaCl, 25 mM HEPES buffer (pH 7.5)  **G4 ligand:** 10 μM NMM (liquid-to-solid transition); 200 μM NMM (aggregates) | U2OS cells, overexpression of wt or mutant LSD1 + TERRA-rG4  **G4 ligand:** NMM (disrupt TERRA-rG4/LSD1 puncta)  **Helicase:** overexpression of DHX36 (reduce telomere clustering) | 116 |  |
| CCND1-dG4/MAZ | **Ionic:** 125 mM NaCl, 50 mM Tris-HCl buffer (pH 7.4), at room temperature  **Crowding agent:** 10% PEG 8000  **G4 ligand:** 0-200 μM TMPyP4; 0-100 μM PDS (reduce CCND1-dG4/MAZ droplet in dose-dependent manner)  **Other:** 10% 1,6-hexanediole (disrupt CCND1-dG4/MAZ condensates) | HCC cells, overexpression of CCND1-dG4 oligo + MAZ wt or mutant  **G4 ligand:** 100 μM TMPyP4; 20 μM PDS (disrupt CCND1-dG4/MAZ puncta) | 28 |  |
| rG4/TDP-43 | **Ionic:** 500 mM KCl, 10 mM Tris-HCl buffer (pH 7.4), at 37°C  **Crowding agent:** 30% PEG 200 | No experimental evidence | 67 |  |
| rG4/FUS | **Ionic:** 150 mM KCl, 0.8 mM MgCl_2,_ 1.8 mM CaCl_2,_ 20 mM PIPES (pH 6.8) | No experimental evidence | 112 |  |
| CGG-rG4/FMRpolyG | **Ionic:** 10 mM MgCl_2,_ 25 mM NaCl, 10 mM Tris-HCl buffer (pH 7.5)  **G4 ligand:** 50 μM PPIX (disrupt CGG-rG4/FMRpolyG aggregates) | FXTAS model mice,  **Other:** 3 mg/kg 5-ALA disrupt CGG-rG4/FMRpolyG aggregates | 30 |  |
| rG4/poly-L-lysine | **Ionic:** 150 mM KCl, 40 mM Tris-HCl buffer (pH 7.5)  **Other:** 4 M urea (solid-to-liquid transition) | No experimental evidence | 119 |  |
| TERRA-rG4/α-syn | | **Ionic:** 140 mM KCl, 10 mM MgCl_2_, 150 mM NaCl, 2 mM CaCl_2_, 50 mM Tris-HCl buffer (pH 7.5), at room temperature  **G4 ligand:** 10 μM PPIX (disrupt TERRA-rG4/α-Syn aggregates)  **Other:** 2.5 mM EGTA (disrupt TERRA-rG4/α-Syn aggregates) | Neuro-2A cells, OptoG4 system;  Mouse model, OptoG4 system  **Other:** 3 mg/kg 5-ALA disrupt TERRA-rG4/α-syn aggregates | 31 |
| rG4/Tau | **Ionic:** 140 mM KCl, 10 mM MgCl_2_, 150 mM NaCl, 2 mM CaCl_2,_ 50 mM Tris-HCl buffer (pH 7.5)  **Crowding agent:** 10% PEG 8000  **Other:** 2.5 mM EGTA (reduce rG4/Tau FRAP level) | HEK-293T cells, rG4/Tau aggregates treatment  **Other:** 500 μM Ca^2+^ (promote rG4/Tau aggregates); 2.5 mM EGTA (reduce rG4/Tau FRAP level) | 120 |  |

Footnotes: For *in vitro* experiments, conditions include physicochemical parameters (e.g., ionic strength, crowding agents, pH, temperature and G4 ligands). For *in cellulo* (or *in vivo*) experiments, conditions include cell line, transfection (e.g., G4-forming oligos and/or protein expression vectors), and treatment (e.g., drugs, G4 ligands, and ions). All references cited in this table are numbered as in the main text.

**Supplemental References**

[S1] N. Sanfeliu-Cerdán, M. Krieg, “The mechanobiology of biomolecular condensates,” *Biophys Rev (Melville)* (2025): 011310, <https://doi.org/10.1063/5.0236610>.

[S2] A. Mangiarotti, R. Dimova, “Phase separation across membranes and condensates in cell organization and function,” *Nat Rev Mol Cell Biol* (2026), <https://doi.org/10.1038/s41580-026-00961-5>.

[S3] N. O. Taylor, M. T. Wei, H. A. Stone, C. P. Brangwynne, “Quantifying Dynamics in Phase-Separated Condensates Using Fluorescence Recovery after Photobleaching,” *Biophys J* (2019): 1285-1300, <https://doi.org/10.1016/j.bpj.2019.08.030>. (This reference is also cited in the main text as ref. 248.)

[S4] D. M. Mitrea, B. Chandra, M. C. Ferrolino, et al., “Methods for Physical Characterization of Phase-Separated Bodies and Membrane-less Organelles,” *J Mol Biol* (2018): 4773-4805, <https://doi.org/10.1016/j.jmb.2018.07.006>.

[S5] S. Dilissen, P. L. Silva, A. Smolentseva, et al., “Characterisation of biocondensate microfluidic flow using array-detector FCS,” *Biochim Biophys Acta Gen Subj* (2024): 130673, <https://doi.org/10.1016/j.bbagen.2024.130673>.

[S6] J. P. Brady, P. J. Farber, A. Sekhar, et al., “Structural and hydrodynamic properties of an intrinsically disordered region of a germ cell-specific protein on phase separation,” *Proc Natl Acad Sci U S A* (2017): E8194-e8203, <https://doi.org/10.1073/pnas.1706197114>.
